# Modeling of the Transport and Exchange of a Gas Species in Lungs With an Asymmetric Branching Pattern. Application to Nitric Oxide

**DOI:** 10.3389/fphys.2020.570015

**Published:** 2020-12-10

**Authors:** Alexandra Buess, Alain Van Muylem, Antoine Nonclercq, Benoit Haut

**Affiliations:** ^1^Transfers, Interfaces and Processes, Ecole Polytechnique de Bruxelles, Université Libre de Bruxelles, Brussels, Belgium; ^2^Chest Department, Erasme University Hospital, Université Libre de Bruxelles, Brussels, Belgium; ^3^Bio-, Electro-, and Mechanical Systems (BEAMS), Ecole Polytechnique de Bruxelles, Université Libre de Bruxelles, Brussels, Belgium

**Keywords:** geometry, model, nitric oxide, exchange, transport, asthma, cystic fibrosis, chronic obstructive pulmonary disease

## Abstract

Over the years, various studies have been dedicated to the mathematical modeling of gas transport and exchange in the lungs. Indeed, the access to the distal region of the lungs with direct measurements is limited and, therefore, models are valuable tools to interpret clinical data and to give more insights into the phenomena taking place in the deepest part of the lungs. In this work, a new computational model of the transport and exchange of a gas species in the human lungs is proposed. It includes (i) a method to generate a lung geometry characterized by an asymmetric branching pattern, based on the values of several parameters that have to be given by the model user, and a method to possibly alter this geometry to mimic lung diseases, (ii) the calculation of the gas flow distribution in this geometry during inspiration or expiration (taking into account the increased resistance to the flow in airways where the flow is non-established), (iii) the evaluation of the exchange fluxes of the gaseous species of interest between the tissues composing the lungs and the lumen, and (iv) the computation of the concentration profile of the exchanged species in the lumen of the tracheobronchial tree. Even if the model is developed in a general framework, a particular attention is given to nitric oxide, as it is not only a gas species of clinical interest, but also a gas species that is both produced in the walls of the airways and consumed within the alveolar region of the lungs. First, the model is presented. Then, several features of the model, applied to lung geometry, gas flow and NO exchange and transport, are discussed, compared to existing works and notably used to give new insights into experimental data available in the literature, regarding diseases, such as asthma, cystic fibrosis, and chronic obstructive pulmonary disease.

## 1. Introduction

**Figure 1:**
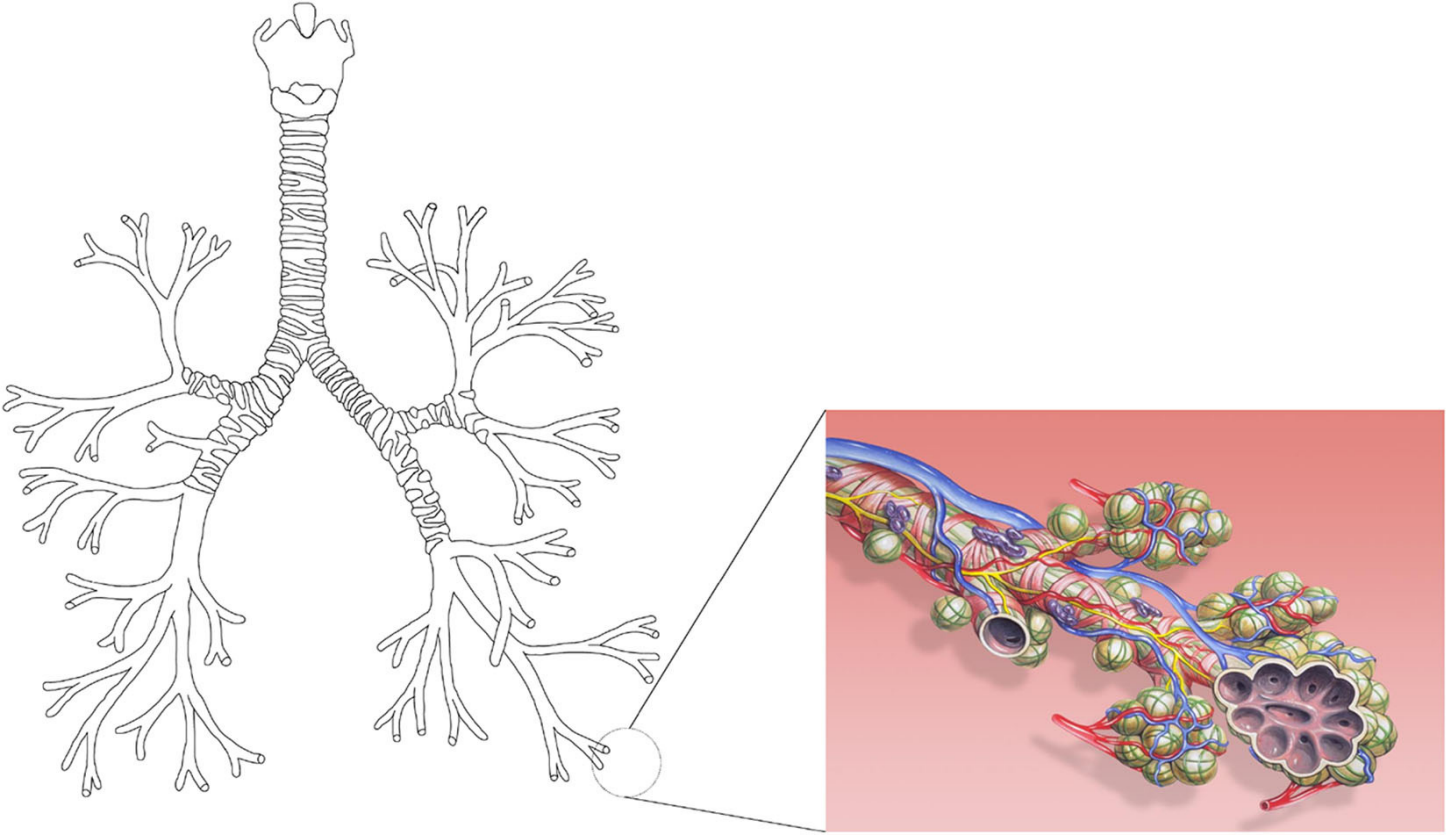
On the left, a schematic representation of the tracheobronchial tree as a dichotomous tubular tree structure (own figure). On the right, a schematic representation of the alveoli on the airway walls of an acinus (Picture by Patrick J. Lynch, medical illustrator/Wikimedia Commons. License: Creative Commons Attribution 2.5 License 2006. Bronchial anatomy detail of alveoli and lung circulation).

With over 10,000 l of air processed a day, the lungs are the body's major exchange site with the environment (Tsuda et al., 2008). Several gas species are exchanged between the lungs and the environment, the most preponderant of them being carbon dioxide, CO_2_, and oxygen, O_2_. The lungs can be divided in two regions, the tracheobronchial tree and the alveolar region. The tracheobronchial tree can be represented as a dichotomous tubular tree structure, starting at the trachea, in which each level is called a generation (see Figure 1). Along the successive generations, the airways become shorter and narrower until the terminal bronchioles are reached. These are the smallest airways without alveoli. The tracheobronchial tree is followed by the alveolar region, composed of acini. Usually, a single acinus is represented as a seven generations dichotomous tree structure, in which each generation has an increasing number of alveoli budding from the walls of its airways (Weibel, 1963; West, 2011). The cells composing the surface of the alveoli form the gas/blood interface between the lungs and the environment.

Over the years, multiple mathematical models have been developed to characterize the transport of a gas species in the lungs and the exchange of this species with the tissues composing this organ. Developing such a model has a dual purpose. First, there is the perpetual strive to gain new insights into the different transport phenomena taking place in the lungs, particularly in the deeper generations where it is extremely difficult to obtain experimental measurements. Indeed, the resolution of medical imaging technologies, such as classic computed tomography or conventional magnetic resonance imaging limits the clear visualization of airways located from the ninth generation on (Lewis et al., 2005; van Ertbruggen et al., 2005; Kleinstreuer and Zhang, 2010; Yablonskiy et al., 2017; Belchi et al., 2018). Most of the models describing the transport and exchange of a gas species in the entire lungs developed so far rely on a homogeneous description of the lung geometry. In such a description, all the airways belonging to a same generation have the same diameter and length. A very known homogeneous description of the lung geometry is the seminal Weibel Type A model (Weibel, 1963). Such a representation is widely used for its simplicity, as it makes it possible not to consider all the airways individually in a given generation and therefore to greatly simplify the modeling (Weibel, 1963; Pedley et al., 1970a; Paiva and Engel, 1981; Nowak et al., 2003; Jalal et al., 2016; Karamaoun, 2017; Karamaoun et al., 2018b; Wells et al., 2018). However, in reality, the lungs present a structural heterogeneity. Indeed, at each bifurcation in the tracheobronchial tree, an airway divides into two daughter airways, both of them being smaller than their parent but one of them being larger than the other one (Tawhai et al., 2004; Florens et al., 2011a). This gives the tracheobronchial tree an asymmetric branching pattern. Additionally, the airways can feature local variations of the diameter of their lumen due to pathological conditions encountered in pulmonary diseases, such as asthma, cystic fibrosis (CF) or Chronic Obstructive Pulmonary Disease (COPD). The number of works considering individually all the airways in the entire tracheobronchial tree is restricted. For instance, Florens et al. (2011a,b) studied the convective transport of oxygen to the end of the tracheobronchial tree in a general lung geometry, based on an asymmetric branching pattern. Herrmann et al. (2016) developed a computational model to analyse the regional gas transport in heterogeneous canine lungs during ventilation. On the other hand, various computational fluid dynamic studies have been carried out, considering the asymmetric character of the tracheobronchial tree, or the transport in the acini. However, these studies often include subject-specific geometries based on medical imaging, limited to a few generations (van Ertbruggen et al., 2005; Lin et al., 2007; Gemci et al., 2008; Katz et al., 2017). The second purpose for developing a mathematical model of the transport and exchange of a gas species in the lungs is driven by the possibility to use it to give new insights into clinical data. Indeed, when a mathematical model is able to reproduce in a qualitative way clinical data obtained in a specific situation, the model can provide additional information for the understanding of the underlying phenomena (see for instance Karamaoun et al., 2018a for such a comparison).

In this work, we present a new multi-scale computational model describing the transport and exchange of a gas species in the lungs. We consider the general case of a species which is both produced and consumed in the lungs. It is mixed with other inert gaseous components, within which it can diffuse and be transported by convection. This model relies on a geometry described by an asymmetric branching pattern. Each airway is considered individually in the model; it allows local alterations of the lungs to mimic situations encountered in certain respiratory diseases. It is a multi-scale model because the phenomena taking place in the wall of the airways as well as in their lumen are considered. The model includes (i) a method to generate a lung geometry characterized by an asymmetric branching pattern, based on the values of several parameters that have to be given by the model user, and a method to possibly alter this geometry to mimic lung diseases, (ii) the calculation of the gas flow distribution in this geometry during inspiration or expiration (taking into account the increased resistance to the flow in airways where the flow is non-established), (iii) the evaluation of the exchange fluxes of the gaseous species of interest between the tissues composing the lungs and the lumen, and (iv) the computation of the concentration profile of the exchanged species in the lumen of the tracheobronchial tree.

The model is presented in this article for the case of nitric oxide, NO, because this molecule is produced and consumed in the lungs (i.e., it represents the most general case). But this is only formal, applying the model to another species, such as O_2_ or CO_2_ just amounts to changing the values of several parameters that appear in the model. For example, regarding O_2_, we would notably zero its production in the lungs and we would modify its equilibrium concentration in the alveolar sacs. NO is preponderantly produced in the epithelial cells of the wall of the airways and consumed in the capillaries surrounding the alveoli. It acts as a bronchodilator by relaxing the smooth muscles of the tracheobronchial tree (Lane, 2004). Additionally, it has been recognized that the molar fraction of this molecule in the exhaled air at the end of an expiration, called the F_E_NO, is increased in numerous pulmonary diseases in which inflammation plays an important role (George, 2008). In asthma, it has been proposed to use the F_E_NO, for an expiration at a flow rate of 50 ml/s, to monitor airway inflammation (American Thoracic Society and European Respiratory Society, 2005). On the other hand, recently, several studies have shown that, in the case of asthma, CF and COPD, the F_E_NO could also be a biomarker for changes in airway caliber (Verbanck et al., 2008; Haccuria et al., 2014; Michils et al., 2016; Karamaoun et al., 2018a; Perez-Bogerd et al., 2019). Previously, different models of the transport and exchange of NO in the lungs, based on a two-compartment approach or on a homogeneous lung geometry, have been developed and used to give new insights into clinical data (Van Muylem et al., 2003; George et al., 2004; Kerckx et al., 2008). For instance, Karamaoun et al. (2016, 2018a) successfully used their model to highlight the sensitivity of the F_E_NO on airway caliber changes. Yet, in reason of the heterogeneous character of lung pathologies, such as asthma, CF, or COPD, the use of such models to give new insights into clinical data is restricted. Consequently, and this is another motivation to present our new model for the case of NO, there is a real need, regarding NO, to develop a model of transport and exchange in the lungs in which it is possible to generate local alterations of the airways to mimic situations encountered in certain respiratory diseases.

What follows is the presentation of our new computational model of the transport and the exchange of a gas species in lungs with an asymmetric branching pattern. The resolution of the equations of the model is also described. Then, several features of the model, applied to lung geometry, gas flow and NO exchange and transport, are discussed, compared to existing works and notably used to give new insights into experimental data available in the literature, regarding diseases, such as asthma, CF, and COPD. It is important to insist on the fact that this work is above all a modeling work. It should be kept in mind that the purpose of a model, such as the one developed in this paper is not to “match” experimental data (in the sense that it is usually understood in engineering sciences, such as fluid mechanics for example). Indeed, due to the fact that the geometry of the lungs is very person-dependent and described in our model by many parameters (see next section), this would require feeding the model with a large number of data, acquired with medical imaging technologies, relating to the lung geometry of the different people involved in the experiments. However, the model must be able to qualitatively reproduce such data and, above all, to highlight interesting phenomena, in particular to shed new light on clinical data/experimental observations. In this sense, the model we are building here and the use we make of it is quite in line with many previous works (Van Muylem et al., 2003; Tawhai and Hunter, 2004; Kerckx et al., 2008; Warren et al., 2010; Florens et al., 2011a; Karamaoun et al., 2018a,b; Wells et al., 2018; Noel and Mauroy, 2019; Park et al., 2019).

## 2. Mathematical Model

In this section, we present the different elements of our computational model. First, we present the way the lung geometry is generated and how it can be subsequently altered to mimic lung diseases. Then, we show how the gas flow distribution in the lungs is modeled and calculated. Lastly, the modeling and the simulation of the transport and the exchange of a gas species are presented. The model is written in a Wolfram Mathematica file, which is made available as Supplementary Material (with full comments). We would like to highlight that the model, although presented here in a dimensional form, has been partially constructed with a dimensionless approach in the Mathematica file, to reduce the computational time. To ease the understanding and lecture of this section, some parts on the mathematical description of the model are detailed in the Appendices. These are available as Supplementary Material.

### 2.1. Geometrical Representation of the Lungs

The model considers a parametrized geometrical representation of the lungs, composed of two regions, the tracheobronchial tree and the alveolar region. As shown below, this geometrical representation includes several parameters. Reference values of these parameters, derived from Weibel (1963) and Florens et al. (2011a), are given in Table 1. As in many previous works, it is assumed that, throughout a respiratory cycle, all sizes defining the geometry of the tracheobronchial tree are constant. Regarding the transport and exchange of NO, this assumption has been validated by Karamaoun et al. (2016).

**Table 1:** Data used for the different parameters defining the reference lung geometry with an asymmetric branching pattern.

**Trachea**	*D*_1,1_	1.5 × 10^−2^ m			
	***L*_1,1_**	**12 × 10^−2^m**			
**Tracheobronchial tree**	*i*	$\chi_{i}^{\text{maj}}$	$\chi_{i}^{\text{min}}$	α_*i*_	σ_*i*_
	1	0.08	0.10	8.00	0
	2	0.01	0.12	3.07	0
	3	0.04	0.12	1.75	0
	4	0.07	0.05	1.43	0
	5	0.08	0.12	1.85	0.02–0.1
	6–…	0.08	0.12	3.00	0.02–0.1
**Terminal airway**	*D*_lim_	2 *d*_alv_			
**Acinus**	*d*_alv_	200 × 10^−6^ m			
	*L*_*a*_	1 × 10^−3^ m			
	*D*_*a*_	400 × 10^−6^ m			
	*n*_alv,1_ = 12,	*n*_alv,2_ = 26,	*n*_alv,3_ = 42,	*n*_alv,4_ = 87,	*n*_alv,5_ = 102,
	*n*_alv,6_ = 123	*n*_alv,7_ = 123			

*Data derived from Weibel (1963) and Florens et al. (2011a)*.

#### 2.1.1. Tracheobronchial Tree

**Figure 2:**
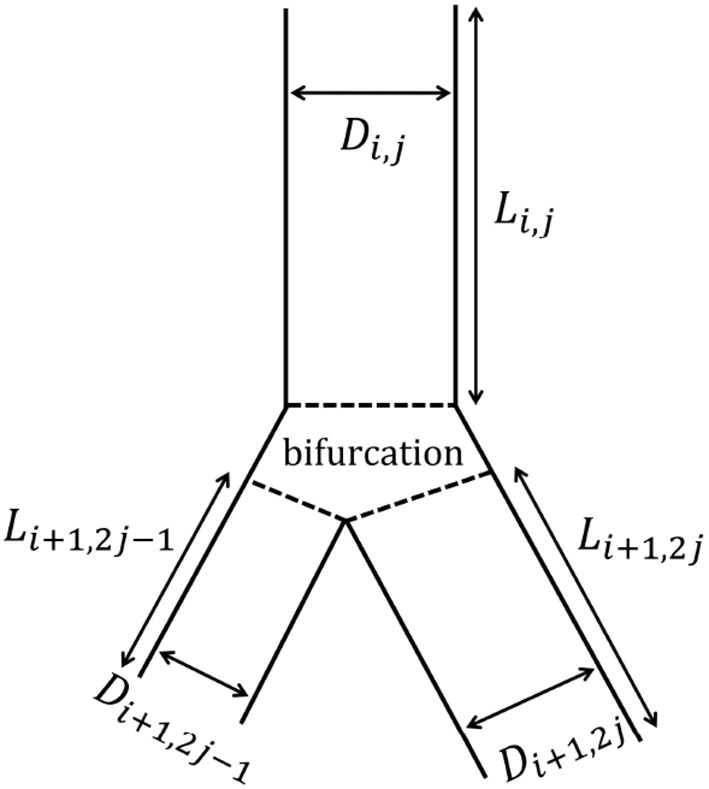
Schematic representation of the branching asymmetry. The airway (*i, j*) gives rise to two daughter airways: the minor airway (*i* + 1, 2*j* − 1) and the major airway (*i* + 1, 2*j*). Both of them are smaller than the airway (*i, j*), but the major one is larger than the minor one.

The tracheobronchial tree is represented as a dichotomous tree. Each airway of the tree is referred to with a couple (*i, j*), where *i* is the generation index and *j* is the airway index within this generation. The trachea is the airway (1, 1). Each airway (*i, j*) divides into two daughter airways, (*i* + 1, 2*j* − 1) and (*i* + 1, 2*j*) (see Figure 2). The airway (*i, j*) is represented as a right circular cylinder of length *L*_*i,j*_ and inner diameter *D*_*i,j*_ (i.e., *D*_*i,j*_ is the diameter of the lumen of the airway).

Our model considers a systematic branching asymmetry for all the generations of the tracheobronchial tree: each parent airway divides into two daughter airways, both of them being smaller than their parent but one of them, called the major airway, being larger than the other one, called the minor airway (see Figure 2). On top of this and as described below, the model also includes the possibility to mimic the inherent local anatomical variability.

In our model, the geometry of the tracheobronchial tree is defined by three scaling ratios depending on the generation index *i*: α_*i*_, $h_{i,0}^{\text{min}}$, and $h_{i,0}^{\text{maj}}$. α_*i*_ is the ratio of the length of an airway in generation *i* to its diameter (see Equation 1). $h_{i,0}^{\text{min}}$ is the ratio of the diameter of a minor airway (belonging to generation *i* + 1) to its parent diameter, in the absence of anatomical variability. $h_{i,0}^{\text{maj}}$ is the ratio of the diameter of a major airway (belonging to generation *i* + 1) to its parent diameter, in the absence of anatomical variability. The value of $h_{i,0}^{\text{min}}$ and $h_{i,0}^{\text{maj}}$ and, therefore, the extent of the systematic branching asymmetry, are given by Equations (2) and (3). $\chi_{i}^{\text{min}}$ and $\chi_{i}^{\text{maj}}$ appearing in these equations are called the asymmetry parameters. In the case of a lung geometry with a symmetric branching pattern, these equations are in accordance with the well-known Hess-Murray law (Sherman, 1981; Mauroy et al., 2004; Florens et al., 2011a). Indeed, if $\chi_{i}^{\text{min}}=\chi_{i}^{\text{maj}}=0$, then $h_{i,0}^{\text{min}}$ = $h_{i,0}^{\text{maj}}$ = 2^−1/3^.

(1) $$\begin{array}{l} L_{i,j}=\alpha_{i}D_{i,j} \end{array}$$

(2) $$\begin{array}{l} h_{i,0}^{\text{min}}=2^{-\frac{1}{3}}-\chi_{i}^{\text{min}} \end{array}$$

(3) $$\begin{array}{l} h_{i,0}^{\text{maj}}=2^{-\frac{1}{3}}+\chi_{i}^{\text{maj}} \end{array}$$

Regarding the inherent local anatomic variability, Florens et al. (2011a) have shown that an anti-correlation between the diameters of the two daughters of a parent airway is able to reproduce at best the morphometric data reported in literature. This is described by Equations (4) and (5), where $h_{i,j}^{\text{min}}$ and $h_{i,j}^{\text{maj}}$ are the actual (i.e., taking into account the local anatomical variability) ratios of the diameters of the minor and major daughters of the airway (*i, j*) to their parent diameter (see Equations 6 and 7). *X*_*i,j*_ appearing in Equation (4) is the outcome of a Gaussian random variable for airway (*i, j*), centered on 0 and with a standard deviation of 1. σ_*i*_ is the dispersion of $h_{i,j}^{\text{min}}$ around its mean value, $h_{i,0}^{\text{min}}$. Equation (5) ensures that the total volume of the daughter airways is conserved despite a randomization of their size.

(4) $$\begin{array}{l} h_{i,j}^{\text{min}}=h_{i,0}^{\text{min}}+\sigma_{i}X_{i,j}\left(0,1\right) \end{array}$$

(5) $$\begin{array}{l} {\left(h_{i,j}^{\text{maj}}\right)}^{3}+{\left(h_{i,j}^{\text{min}}\right)}^{3}={\left(h_{i,0}^{\text{maj}}\right)}^{3}+{\left(h_{i,0}^{\text{min}}\right)}^{3} \end{array}$$

(6) $$\begin{array}{l} D_{i+1,2j-1}=h_{i,j}^{\text{min}}D_{i,j} \end{array}$$

(7) $$\begin{array}{l} D_{i+1,2j}=h_{i,j}^{\text{maj}}D_{i,j} \end{array}$$

The tracheobronchial tree is generated until an ending condition is met. When an airway (*i, j*) generates at least one daughter airway that has a diameter that is smaller than or equal to a critical diameter, *D*_lim_, both of the daughter airways of this airway (*i, j*) are not generated. If this condition is met, airway (*i, j*) is said to be a terminal airway. *D*_lim_ is a parameter of the model and we assume that it is equal to twice the inner diameter of an alveolus, *d*_alv_ (Weibel, 1963; Florens et al., 2011a; Karamaoun, 2017):

(8) $$\begin{array}{l} D_{\text{lim}}=2d_{\text{alv}} \end{array}$$

A is the set of all the airways composing the tracheobronchial tree. T is the set of the terminal airways. The number of terminal airways is written *n*_term_, while the total number of airways in A is written *n*_airw_. It can easily be demonstrated that *n*_airw_ = 2*n*_term_ − 1. The airways belonging to B=A-T are called the intermediate airways.

#### 2.1.2. Alveolar Region

The alveolar region is characterized by the presence of acini. It is assumed that two acini are connected to each terminal airway (Weibel, 1963). An acinus is represented as a dichotomous tree structure, composed of seven generations. The generation index within an acinus is written *m* (*m* = 1, …, 7). As in the work of Noel and Mauroy (2019), we assume that all the airways within an acinus are right circular cylinders of length *L*_*a*_ and diameter *D*_*a*_. This is a simplifying assumption in view of the fact that, in a real acinus, there is a small reduction of the diameter of the airways along successive generations. However, this reduction is much smaller than the one in the tracheobronchial tree (Weibel, 1963; Park et al., 2019). It has been verified that the results obtained with our model, using the assumption of constant length and diameter of the airways in the acini, show no observable difference with results obtained using a model in which an appropriate reduction factor is introduced. Along an acinus, the amount of alveoli budding on the walls of the airways progressively increases. The total number of alveoli in an airway in generation *m* of an acinus is written *n*_alv,*m*_. It is evaluated using the morphological data of Weibel (1963).

#### 2.1.3. Flexibility of the Geometrical Representation

It is worth highlighting the flexibility of our parametrized geometrical representation. By specifying values of α_*i*_, $\chi_{i}^{\text{min}}$, $\chi_{i}^{\text{maj}}$, σ_*i*_ (all dependent on the generation index *i*), and *D*_1,1_, the model allows defining a complex lung geometry of an adjustable size. Moreover, after the generation of the lung geometry, the geometrical parameters of all the airways [i.e., *L*_*i,j*_ and *D*_*i,j*_, ∀(i,j)∈A] are stored individually in the Wolfram Mathematica file. Therefore, as presented in the results section, the properties of some airways can be selectively modified to mimic situations encountered in certain respiratory diseases. Additionally, as presented below, two parameters are assigned to each terminal airway, to include the possibility to introduce some defects in its two attached acini (swelling, accumulation of liquid, lack of perfusion…). These elements are reflected in the Wolfram Mathematica file by various functions that have been defined to allow the user of the model to carry out local alterations of the lungs (regarding the airways in the tracheobronchial tree or the acini attached to the terminal airways).

### 2.2. Gas Flow in the Tracheobronchial Tree

In the previous section, it is shown how the geometrical representation of the lungs is generated. The next step of the model consists in calculating the gas flow rate, *Q*_*i,j*_, and the average axial gas velocity, $V_{i,j}=4Q_{i,j}/\left(\pi D_{i,j}^{2}\right)$, in each airway (i,j)∈A, for a given flow rate in the trachea or alveolar pressure (possibly time-dependent). As this calculation is performed using a classical electrical analogy, it is not fully detailed in the core of the paper, but in Appendix 1. Two elements of this modeling are however worth to be described in the core of the text.

First, within the framework of this electrical analogy, a so-called “peripheral” or “subtree” flow resistance, written $R_{i,j}^{\text{sub}}$, is defined for each airway (*i, j*). It is the resistance to the flow exhibited by the whole part of the lungs that is downstream of the airway. For a terminal airway [i.e., for (i,j)∈T], this subtree resistance is the resistance to the flow of the two acini, in parallel, located after this terminal airway:

(9) $$\begin{array}{l} R_{i,j}^{\text{sub}}=\frac{1}{2}\frac{R_{a}}{\phi_{i,j}^{3}} \end{array}$$

with $R_{a}$ the resistance to the flow in an acinus. ϕ_*i,j*_ is a parameter whose value is set to 1 when the lung geometry is generated by the model [∀(i,j)∈T]. However, by using some of the “alteration functions” mentioned above, the user of the model can later reduce its value to a number strictly between 0 and 1 for some terminal airways. This allows to mimic the effect of alterations in the acini downstream of these airways (swelling, accumulation of liquid…) on the resistance to the flow in these acini. ϕ_*i,j*_ is set to the power of three in the denominator on the right-hand side of Equation (9) so that ϕ_*i,j*_ represents a reduction factor of the linear dimensions of the lumen of the acini downstream of the airway (*i, j*).

Assuming a Poiseuille flow in each generation of an acinus, as the Reynolds number of the flow in an airway of an acinus is usually smaller than 1, $R_{a}$, is calculated as:

(10) $$\begin{array}{l} R_{a}=\overset{7}{\underset{m\;=\;1}{\sum }}\frac{1}{2^{m-1}}\frac{128\rho \nu \;L_{a}}{D_{a}^{4}}=254\frac{\rho \nu L_{a}}{D_{a}^{4}} \end{array}$$

with ρ the density of the gas and ν its kinematic viscosity.

Second, in our approach, we take into account the fact that, depending on the position in the tracheobronchial tree and the inspiratory/expiratory flow rate, the flow in an airway may be far from established (i.e., significantly different from the Poiseuille parabolic flow). This results in an increase of the resistance to the flow in this airway, in comparison with the Poiseuille resistance. To account for this in our model, we evaluate the resistance to the flow in the airway (*i, j*) as the Poiseuille resistance multiplied by a factor *Z*_*i,j*_ ≥ 1, depending on the Reynolds number of the flow in the airway (see Appendix 1 for the expression of this factor). Consequently, as the resistances thus depend on the flow rates, the electrical analogy generates a system of non-linear equations to describe the flow distribution in the entire tracheobronchial tree. As described in the Appendix 1, we have set up, in the Wolfram Mathematica file, a simple procedure for an iterative resolution of this system of equations.

### 2.3. NO Exchange and Transport in the Lungs

#### 2.3.1. Exchange of NO Between the Tissues and the Lumen

As mentioned earlier, NO is produced in the epithelium of the airways and is consumed by the blood capillaries surrounding these airways and the alveoli. Therefore, it is necessary to establish two equations to describe the exchange of NO between the tissues and the lumen: an equation describing these exchanges in the tracheobronchial tree and another describing it in the acinus. If a measurement of the concentration of NO in the exhaled air (F_E_NO test) is to be simulated by the model, these two exchanges must be included in the model. Indeed, during such a test, the production of NO in the walls of the airways and its consumption in the alveoli are both significant. On the other hand, if the model has to simulate the measurement of the pulmonary diffusing capacity of NO (DLNO), then the exchange between the bronchial walls and the lumen should not be included in the model, given that the large amount of inhaled NO during such a test marginalizes its production in the walls of the bronchi.

Table 2 summarizes the values used in the results section for the different physicochemical and physiological parameters involved in the equations derived below.

**Table 2:** Overview of the physicochemical and physiological parameters involved in the modeling of NO transport and exchange in a healthy human.

**Parameter**	**Value**	**Units**
ν	1.67 × 10^−5^	m^2^s^−1^
ρ	1.14	kgm^−3^
$D_{g}$	2.2 × 10^−5^	m^2^s^−1^
$D_{t}$	3.3 × 10^−9^	m^2^s^−1^
*k*_*t*_	0.2	s^−1^
*k*_alv_	2 × 10^−3^	m s^−1^
*P*	25 × 10^−6^	molNOm^−3^s^−1^
*C*_*eq*_	2	ppb
δ_μ_	10 × 10^−6^	m
δ_*E*_	10 × 10^−6^	m
δ_*M*_	30 × 10^−6^	m

*Data derived from Malinski et al. (1993), Vaughn et al. (1993), Tsoukias and George (1998), Sobac et al. (2015), and Karamaoun et al. (2016) (at atmospheric pressure, at 37°C and at a relative humidity of 1)*.

**Figure 3:**
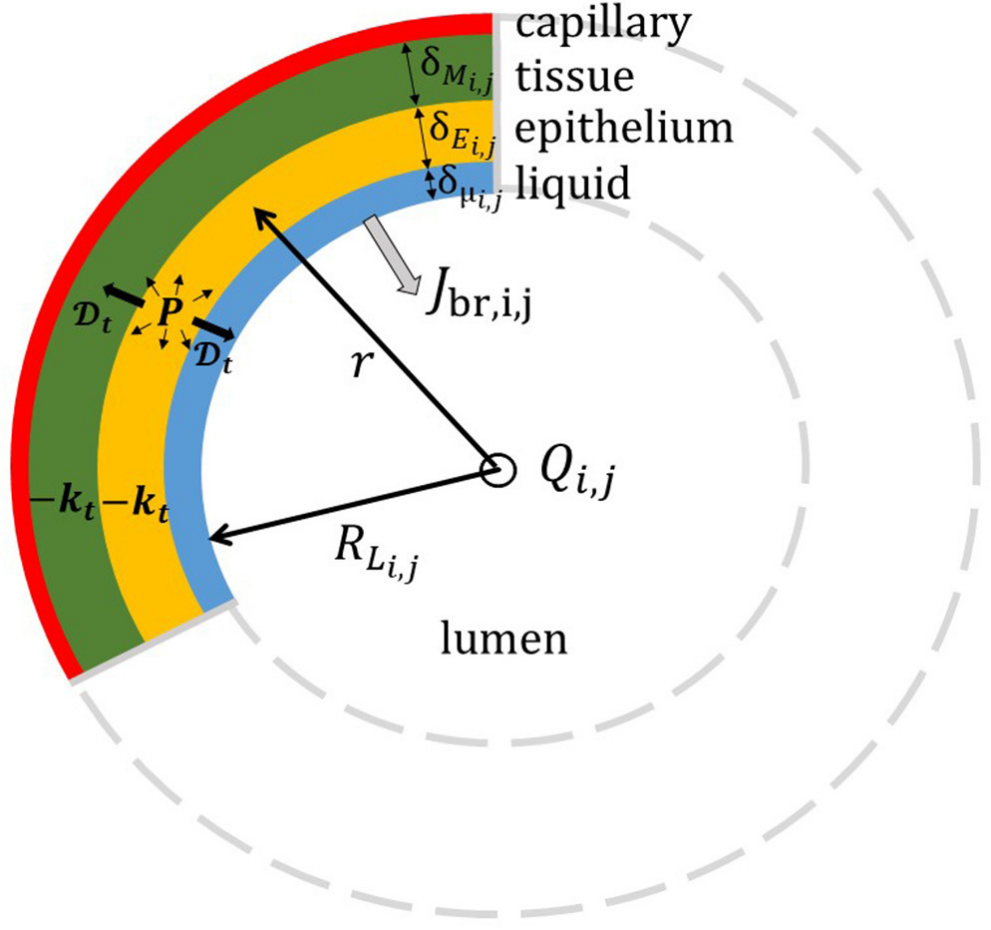
Schematic representation of the cross-section of the airway (*i, j*) in the tracheobronchial tree. The wall of the airway is represented as a layered structure surrounding the lumen, composed of a liquid layer, an epithelium layer and a tissue layer, with a thickness δ_μ_*i,j*__, δ_*E*_*i,j*__, and δ_*M*_*i,j*__, respectively. *R*_*L*_*i,j*__ = *D*_*i,j*_/2 is the radius of the lumen. NO is produced in the epithelium and consumed in the epithelium and the tissue layer.

Regarding the exchanges in the tracheobronchial tree, Figure 3 gives a schematic representation of the cross-section of the wall of the airway (*i, j*) in the tracheobronchial tree and of the phenomena taking place within it. The wall of the airway is represented as a layered structure surrounding the lumen, composed of a liquid layer (which in a non-pathological situation is typically the Airway Surface Liquid with a thickness of around 10 μm), an epithelium layer and a tissue layer, with a thickness δ_μ_*i,j*__, δ_*E*_*i,j*__, and δ_*M*_*i,j*__, respectively. According to the location in the lungs, the composition of the tissue layer is not the same. In the upper airways, the tissue layer is majorly composed of a connective tissue with blood vessels, called the lamina propria, and cartilageous structures (Alkanli and Koroglu, 2019). From the respiratory bronchioles on (i.e., the airways with an average diameter less than 0.5 mm), this tissue is mainly built up by smooth muscle. The tissue layer is surrounded by a dense network of blood capillaries (Weibel, 1963). As in Vaughn et al. (1993), we assume that the diffusion coefficient of NO in these three layers, written $D_{t}$, is the same. According to the results of Malinski et al. (1993), we use $D_{t}=3.3$ 10^−9^ m^2^/s. The volumetric production rate of NO is written *P*. It is evaluated using the results presented in Vaughn et al. (1993). The transfer flux density of NO from the bronchial wall into the lumen of the airway (*i, j*) is written *J*_br,*i,j*_. It gives the amount of NO penetrating into the lumen, per unit area of the liquid-lumen interface and per unit of time.

When a NO molecule is produced in the epithelial layer, it has three different options. First, a NO molecule can diffuse through the epithelial layer and the tissue layer to reach the blood capillaries where it is consumed. Second, a NO molecule can diffuse through the epithelial layer and the liquid layer to reach the lumen. Lastly, it is also possible for a NO molecule to be consumed within the epithelial or the tissue layer (Beckman and Koppenol, 1996; Karamaoun et al., 2016). Indeed, within these layers, several reactions with NO take place, such as the reaction with superoxide to form peroxynitrite (Beckman and Koppenol, 1996; Rajendran et al., 2019). These reactions were shown to be well-represented by a first-order reaction with respect to the NO concentration (Beckman and Koppenol, 1996). It is assumed that the kinetic constant of this first-order reaction, *k*_*t*_, is independent of the type of layer (epithelium or tissue). Its value is taken from Tsoukias and George (1998).

It should be noted that this representation of the wall of an airway and of the phenomena taking place within its different layers give a better picture of the distal airways than of the proximal airways. However, as shown later, this has no influence on the results as the distal airways have a far more important impact on the NO exchange in the tracheobronchial tree than the proximal ones.

As detailed in Appendix 2, a set of equations is written to describe this representation of the phenomena taking place in the wall of the airway. This system relies on different assumptions. Notably, we assume that the NO concentration can be set to zero at the interface between the tissue and the blood (due to its high affinity for NO) and in the liquid at the liquid-lumen interface. This last assumption is motivated by the fact that the NO concentrations in water at equilibrium with a gas containing NO at typical concentrations of F_E_NO tests are much lower than those generated in the epithelium by the production of NO and its diffusion into it. An important consequence of these two assumptions is that *J*_br,*i,j*_ is thus time-independent. Manipulating the system of equations presented in Appendix 2 allows proposing the following approximate expression for *J*_br,*i,j*_:

(11) $$\begin{array}{l} J_{\text{br},i,j}=\frac{P\delta_{E_{i,j}}}{2}\frac{\delta_{E_{i,j}}+2\delta_{M_{i,j}}}{\delta_{\mu_{i,j}}+\delta_{E_{i,j}}+\delta_{M_{i,j}}+\frac{\delta_{\mu_{i,j}}}{2}{\text{Ha}}_{i,j}^{2}} \end{array}$$

In this equation, the transfer flux density is logically proportional to the epithelial NO production rate *P* and depends on the thickness of the different layers composing the wall of the airway. It also depends on a dimensionless Hatta number: ${\text{Ha}}_{i,j}=\left(\delta_{E_{i,j}}+\delta_{M_{i,j}}\right)\;\sqrt{k_{t}/D_{t}}$. It compares a characteristic time of NO consumption in the wall of the airway to a characteristic time of transport by diffusion within this wall. If this number is large compare to 1, the NO flux delivered into the lumen is significantly reduced because of the high consumption of NO in the tissues.

The total amount of NO delivered per unit of time in the airway (*i, j*), written *F*_*i,j*_, is obtained by multiplying *J*_br,*i,j*_ by the area of the lateral surface of the airway:

(12) $$\begin{array}{l} F_{i,j}=\pi L_{i,j}D_{i,j}J_{\text{br},i,j} \end{array}$$

For a healthy human, we assume that the physicochemical and physiological parameters appearing in the expression of *J*_br,*i,j*_ (and thus *F*_*i,j*_) are constant throughout the tracheobronchial tree. Therefore, when the lung geometry is generated by the model, constant values are assigned to δ_μ_*i,j*__, δ_*E*_*i,j*__, and δ_*M*_*i,j*__: δ_μ_*i,j*__ = δ_μ_, δ_*E*_*i,j*__ = δ_*E*_ and δ_*M*_*i,j*__ = δ_*M*_. However, by using some of the “alteration functions” mentioned above, the user of the model can later modify selectively their values, according to different scenarios presented in section 3.2.1, notably by the introduction of a factor β, defined in Equation (19), measuring the reduction of the lumen area due to these alterations.

Regarding the exchanges in the acini, we write *J*_*a,m*_ the amount of NO delivered to the lumen of an airway in generation *m* of an acinus, per unit of time and per unit area of the theoretical lateral surface of the airway, π*D*_*a*_*L*_*a*_. Within an acinus, the amount of alveoli budding on the lateral wall of an airway, *n*_alv,*m*_, is increasing with the generation number *m*. Consequently, the area of the available lateral wall surface is progressively decreasing and eventually becomes zero when an airway is completely covered by these grape-like alveolar openings. In an alveolus, two layers of cells form a barrier, through which gas molecules diffuse, between the lumen and the blood capillaries. This alveolar epithelium is around 2 μm thick (Knudsen and Ochs, 2018). In the model, an alveolus is represented by half a sphere, of inner diameter *d*_alv_. The lateral walls of the airways of an acinus are composed of an epithelium layer of thickness δ_*E*_ and a tissue layer of thickness δ_*M*_, surrounded by capillaries acting as an infinite sink.

In our model, *J*_*a,m*_ is expressed as:

(13) $$\begin{array}{l} J_{a,m}=-\gamma k_{\text{alv}}\left(C_{g,m}-C_{\text{eq}}\right)\frac{n_{\text{alv},m}d_{\text{alv}}^{2}}{2D_{a}L_{a}}+\frac{P\delta_{E}}{2}\frac{\delta_{E}+2\delta_{M_{i,j}}}{\delta_{E}+\delta_{M}}\\ \left(1-\frac{n_{\text{alv},m}d_{\text{alv}}^{2}}{4D_{a}L_{a}}\right) \end{array}$$

The first term on the right-hand side of this equation expresses the amount of NO penetrating the thin barrier of the alveoli to react with hemoglobin in the blood, per unit of time and per unit area of the alveolar surface, multiplied by the ratio of the area of the alveolar surface in generation *m* to the area of the theoretical lateral surface of this generation, $n_{\text{alv},m}d_{\text{alv}}^{2}/\left(2D_{a}L_{a}\right)$. The minus sign in front of this term is such that this flux has the same direction as *J*_br,*i,j*_ (i.e., positive when NO is transferred from the alveolar epithelium toward the lumen and negative if NO diffuses from the lumen toward the blood through the alveolar epithelium). *C*_*g,m*_ is the local NO concentration in the lumen in generation *m* of the acinus, *C*_eq_ is the NO concentration in equilibrium with the blood and *k*_alv_ is the ratio of the diffusion coefficient of NO in the alveolar tissue to the thickness of the alveolar epithelium. γ is called the perfusion coefficient of the acinus. By default, γ is taken equal to 1 but, as detailed below, the user of the model can lower its value to a number ∈[0, 1] for some acini, giving the possibility of introducing local perfusion defects. Regarding NO, the mechanism by which changes to the perfusion are achieved is predominated by vessel recruitment (Coffman et al., 2017).

The second term on the right-hand side of Equation (13) is the amount of NO produced in the airway wall and delivered in the lumen per unit of time and per unit area of the theoretical lateral wall surface (assumed given by Equation 11, with δ_μ_*i,j*__ = 0, as there is no liquid in a healthy acinus, δ_*E*_*i,j*__ = δ_*E*_ and δ_*M*_*i,j*__ = δ_*M*_), multiplied by a factor, $1-n_{\text{alv,m}}d_{\text{alv}}^{2}/\left(4D_{a}L_{a}\right)$, accounting for the reduction of the area of the airway wall due to the presence of the alveoli. This factor is set to zero if it is initially evaluated negative.

#### 2.3.2. Transport of NO in the Lumen

The transport of NO in the lumen is modeled in two parts: the transport in a single acinus and the transport in the airways of the tracheobronchial tree.

The transport of a gas species in the human acini has been the subject of many previous studies, notably considering 3D complex structures (Felici et al., 2005; Hofemeier et al., 2016). Here, we use a classical quasi-steady 1D approach, as in several works (Paiva, 1972, 1973; Karamaoun et al., 2016; Noel and Mauroy, 2019). It is fully described in Appendix 3. Briefly, a system of equations, composed of a convection-diffusion equation written for each generation of the acinus and of appropriate boundary conditions, is written. This system can be solved analytically, in order to provide a relation (not explicitly given here due to its prohibitive size) between the concentration gradient at the proximal end of an acinus (i.e., its inlet during inspiration or its outlet during expiration), the perfusion coefficient γ of the acinus, and the NO concentration and gas flow rate at this proximal end:

(14) $$\begin{array}{l} {\frac{\partial C_{g,1}}{\partial z}|}_{z\;=\;0,t}=f^{\pm }\left(\gamma ,C_{g,1}\left(0,t\right),Q_{a}\left(t\right)\right) \end{array}$$

where *z* is an axial coordinate in the first airway of the acinus, oriented in the direction of the alveolar sacs, with *z* equal to zero at the proximal end of the airway and equal to *L*_*a*_ at its distal end, *C*_*g*, 1_(*z, t*) is the NO concentration in the lumen, at time *t* and at position *z* in the first airway of the acinus, and *Q*_*a*_(*t*) is the flow rate of gas entering or leaving the acinus. The sign ± as a superscript accounts for the fact that two versions of the function *f* exist, whether inspiration (+) or expiration (−) is considered.

As described below, Equation (14) serves as a connecting link between the model of the NO transport in an acinus and the model of the NO transport in the tracheobronchial tree. Indeed, it is included in a boundary condition written at the end of each terminal airway.

Regarding the transport in the lumen of the tracheobronchial tree, a 1D unsteady convection-diffusion equation is written for each individual airway (i,j)∈A:

(15) $$\begin{array}{l} \frac{\partial C_{g,i,j}\left(z,t\right)}{\partial t}\pm \;\frac{4Q_{i,j}}{\pi D_{i,j}^{2}}\frac{\partial C_{g,i,j}\left(z,t\right)}{\partial z}=D_{g}\frac{\partial^{2}C_{g,i,j}\left(z,t\right)}{\partial z^{2}}+\frac{4}{D_{i,j}}J_{\text{br},i,j}\\ \;\forall \left(i,j\right)\in A \end{array}$$

where *C*_*g, i, j*_(*z, t*) is the NO concentration in the lumen of the airway (*i, j*) at time *t* and at position *z* in the airway, with *z* an axial coordinate (*z* = 0 at the proximal end of the airway and *z* = *L*_*i,j*_ at its distal end). $D_{g}$ is the diffusion coefficient of NO in the lumen (its value for the diffusion of NO in air is given in Table 2). This transport equation includes an accumulation term and a convective term on its left-hand side, with the ± sign accounting for the direction of the gas flow during inspiration (+) or expiration (−), and a diffusion term and an exchange term on its right-hand side.

These convection-diffusion equations are completed by boundary conditions. According to the type of airway, i.e., entrance, intermediate, or terminal, and the regime of respiration, i.e., inspiration or expiration, different boundary conditions are written:

(16a) $$\begin{array}{l} \text{Trachea}\{\begin{array}{ll} \text{inspiration:} & {C_{g,1,1}|}_{z=0,t}=C_{\text{in}}\;\\ \text{expiration:} & {\frac{\partial C_{g,1,1}}{\partial z}|}_{z=0,t}=0\; \end{array} \end{array}$$

(16b) $$\begin{array}{l} \text{If}\left(i,j\right)\in B\{\begin{array}{l} {C_{g,i,j}|}_{z=L_{i,j},t}={C_{g,i+1,2j-1}|}_{z\;=\;0,t}={C_{g,i+1,2j}|}_{z=0,t}\\ D_{i,j}^{2}{\frac{\partial C_{g,i,j}}{\partial z}|}_{z\;=L_{i,j},t}=D_{i+1,2j-1}^{2}{\frac{\partial C_{g,i+1,2j-1}}{\partial z}|}_{z\;=\;0,t}\\ \;+D_{i+1,2j}^{2}{\frac{\partial C_{g,i+1,2j}}{\partial z}|}_{z\;=\;0,t} \end{array} \end{array}$$

(16c) $$\begin{array}{l} \text{If}\left(i,j\right)\in T\{\begin{array}{l} D_{i,j}^{2}{\frac{\partial C_{g,i,j}}{\partial z}|}_{z=L_{i,j},t}\\ =2{\left(\phi_{i,j}D_{a}\right)}^{2}f^{\pm }\left(\gamma_{i,j},C_{g,i,j}{|}_{z=L_{i,j},t},\frac{Q_{i,j}}{2}\right)\; \end{array} \end{array}$$

At the inlet of the trachea, the concentration is imposed at its atmospheric value (*C*_in_) during an inspiration whereas, for an expiration, the boundary condition at the proximal end of the trachea is the absence of axial diffusion (see Equation 16a).

For an intermediate airway, the two boundary conditions applied, given by Equation (16b), are the continuity of the concentration and the conservation of the NO diffusive flux at the interface between the airway and its two daughters.

For a terminal airway, the conservation of the diffusive flux at its distal end, given by Equation (16c), is expressed using the linking function *f*^±^ described previously. In this equation γ_*i,j*_ is the value of the perfusion coefficient of the two acini downstream of the terminal airway (*i, j*). Similarly to the parameter ϕ_*i,j*_, when the lung geometry is generated by the model, the value of 1 is assigned to the parameter γ_*i,j*_ appearing in Equation (16c), ∀(i,j)∈T. However, by using some of the “alteration functions” mentioned previously, the user of the model can later reduce its value to a number ∈[0, 1] for some terminal airways. It gives the possibility of introducing local perfusion defects, by reducing the consumption of NO in the acini downstream of the terminal airway (*i, j*).

Depending on the type of clinical measurement with which the model must be confronted to, the numerical approach used to solve these transport equations vary. A classic F_E_NO measurement consists in measuring the NO molar fraction in the exhaled air at the end of a 10 s expiration at a flow rate of 50 ml/s (American Thoracic Society and European Respiratory Society, 2005). It has been shown previously that the NO profile in the lungs at the end of such a long expiration is at steady state (Van Muylem et al., 2003). Therefore, when simulating a F_E_NO test, the transport equations in the tracheobronchial airways can be solved with the time derivative of the NO concentration in the transport equations set to zero (see Equation 15). Consequently, for each airway in the tracheobronchial tree, the resulting convection-diffusion transport equation has an analytical solution with two unknown coefficients, *A*_*i,j*_ and *B*_*i,j*_. These unknown coefficients can be determined by introducing the different analytical solutions for *C*_*g, i, j*_(*z, t*) in the boundary conditions stated in Equations (16a)–(16c), yielding a set of 2*n*_airw_ linear equations with 2*n*_airw_ unknowns [*A*_*i,j*_ and *B*_*i,j*_, ∀(i,j)∈A]. On the other hand, when studying time dependent clinical measurements, such as the pulmonary diffusing capacity of NO (DLNO), unsteady conditions apply and the equations have to be solved numerically. For this purpose, a numerical scheme, based on the finite volume method, has been implemented in the Wolfram Mathematica file. The details of this numerical scheme are presented in Appendix 4.

## 3. Results

In this section, several features of the model, applied to lung geometry, gas flow distribution and NO exchange and transport, are discussed. The results obtained with the model are put in parallel with existing works and used to shed new light on the dynamics of NO in unhealthy lungs.

**Figure 4:**
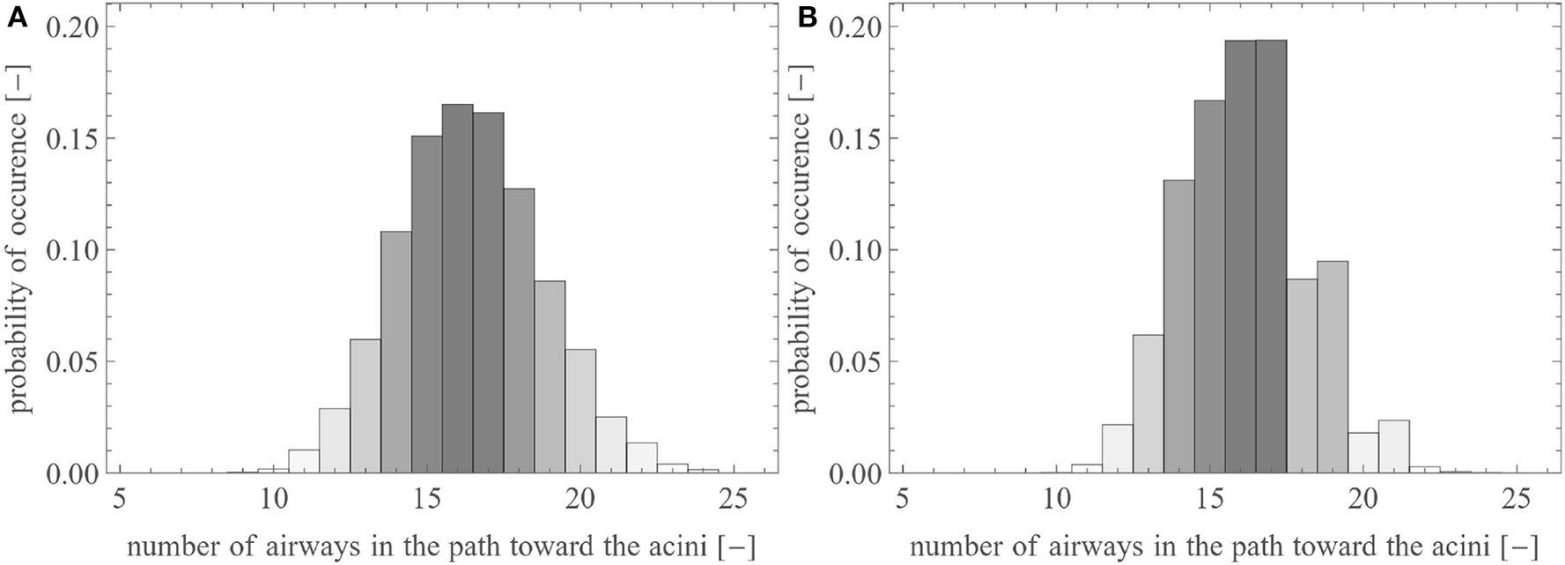
Probability distribution of the number of airways within a pathway toward the acini **(A)** in the reference lungs and **(B)** in the reference lungs without anatomical variability [i.e., with $\sigma_{i}=0,\forall \left(i,j\right)\in A$].

In this section, we use the reference geometry of the human lungs described by the set of parameters presented in Table 1, with σ_*i*_ equal to 0.05 (except for the first four generations, for which σ_*i*_ = 0). For lungs generated with these parameters, Figure 4A shows the probability distribution of the number of airways needed to reach an acinus (i.e., the distribution of the generation index *i* of the terminal airways). The majority of the acini are located after 15–17 successive airways. The total number of terminal airways within the set T is around 15,000, giving rise to 30,000 acini. This representation is in very good accordance with morphometric data available in literature (Weibel, 1963; Horsfield and Cumming, 1968; Florens et al., 2011a).

To illustrate the impact of the inherent local anatomical variability on the distribution of the terminal airways, Figure 4B shows the same results for a lung with the same volume, the same branching asymmetry (i.e., the same values of $\chi_{i}^{\text{min}}$ and $\chi_{i}^{\text{maj}}$), but no variability (i.e., σ_*i*_ is set to zero, see Equation 4). It can be observed that this lack of anatomical variability leads to a sharp decrease in the number of terminal airways after generation 17.

### 3.1. Gas Flow in the Tracheobronchial Tree

#### 3.1.1. Delivery of a Gas Species to the Acini

An important parameter to characterize the competition between the convective and the diffusive transports of a gas species in an airway is the Peclet number. In airway (*i, j*), it is defined as ${\text{Pe}}_{i,j}=V_{i,j}L_{i,j}/D_{g}$, with $D_{g}$ the diffusion coefficient of the considered species in the lumen. This dimensionless number is the ratio of the axial diffusive characteristic time to the axial convective characteristic time in the lumen of the airway. If Pe >> 1, convection dominates diffusion while, if Pe << 1, diffusion dominates convection. Considering a flow rate in the trachea equal to 50 ml/s and using the reference geometry, the model calculates that, in the terminal airways, the Peclet number is 0.70±0.25 (using the diffusion coefficient of NO in air). This indicates that axial convective and diffusive transports are of the same order of magnitude. On the other hand, if a flow rate of 250 ml/s is considered in the trachea, the model calculates that, in the terminal airways, the Peclet number is 3.52±1.29 (still using the diffusion coefficient of NO in air). This indicates that, regarding the axial transport, convection prevails against diffusion in the entire tracheobronchial tree (as, in this tree, the smallest values of Pe are met in the terminal airways).

For respiratory conditions implying Peclet numbers larger than 1 in the entire tracheobronchial tree, it is reasonable to assume that the axial gas transport in the tracheobronchial tree is dominated by convection. Under this assumption, the distribution of the flow rates *Q*_*i,j*_ in the tracheobronchial tree can be linked to the amount of any gas species that is reaching the acini. As an illustrative example, we analyse here the delivery of a gas species to the acini for a constant inspiration flow rate *Q*_1,1_. For this purpose, two parameters are introduced. The first one, given by Equation (17), is the delivery time of the acini beyond the terminal airway (*i, j*), *t*_del_*i,j*__. It is defined as the inspiration time, *t*_ins_, minus the time spent by the gas in the oropharyngeal and laryngeal cavities, *t*_cav_, and minus the transit time needed for the gas to go from the trachea to the end of the terminal airway (*i, j*), *t*_trans_*i,j*__:

(17) $$\begin{array}{l} t_{{\text{del}}_{i,j}}=t_{\text{ins}}-\left(t_{\text{cav}}+t_{{\text{trans}}_{i,j}}\right) \end{array}$$

*t*_cav_ is obtained by dividing the volume of the cavities, around 106 ml, by *Q*_1,1_ (Florens et al., 2011a). The transit time is obtained by summing up the residence times *L*_*k,l*_/*V*_*k,l*_ in each airway (*k, l*) along the pathway from the trachea down to the end of the terminal airway (*i, j*).

The second parameter is the gas supply ratio to the terminal airway (*i, j*), Ω_*i,j*_, defined as the ratio of the amount of the gas species reaching an acinus beyond airway (*i, j*) to the amount of this species that would reach this acinus if *t*_trans_*i,j*__ was zero and if the gas species was delivered equally to all the acini in the lungs:

(18) $$\begin{array}{l} \Omega_{i,j}=\frac{Q_{i,j}t_{{\text{del}}_{i,j}}}{\frac{Q_{1,1}}{n_{\text{term}}}\left(t_{\text{ins}}-t_{\text{cav}}\right)} \end{array}$$

**Figure 5:**
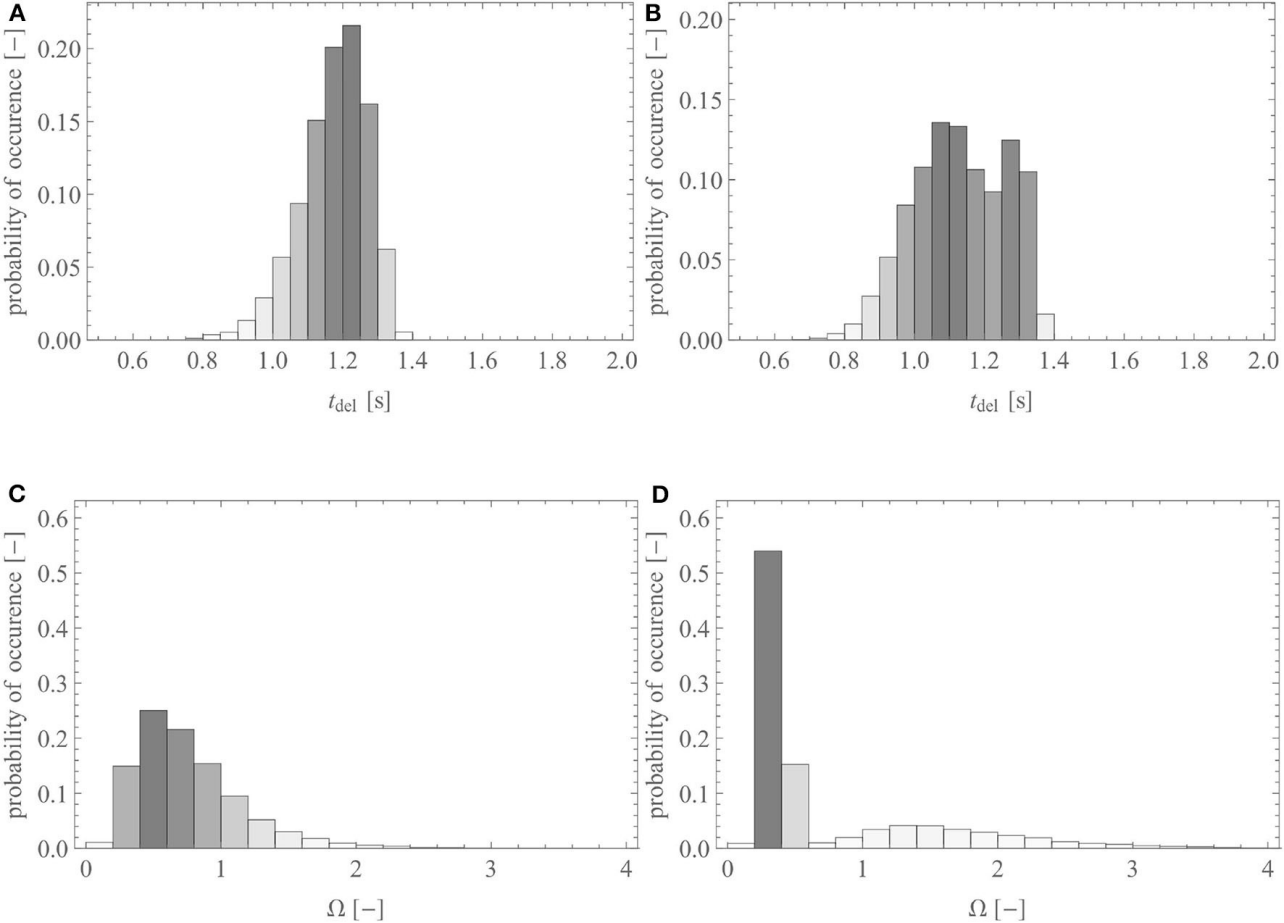
**(A)** Probability distribution of the delivery time in the reference geometry and **(B)** in a pathological scenario where 70% of the acini have a reduction of the inner diameter of all their airways of 50%. **(C)** Probability distribution of the gas supply ratio, Ω, in the reference geometry and **(D)** in a pathological scenario where 70% of the acini have a reduction of the diameter of all their airways of 50%.

Figures 5A,C show the probability distribution of the delivery time and of the gas supply ratio in the reference geometry of the lungs, for *Q*_1,1_ = 250 ml/s and *t*_ins_ = 2 s (typical values for the inspiration at rest for a healthy adult), respectively. It is worth to mention that the distribution presented in Figure 5A is very close to the one presented in the work of Florens et al. (2011a), for the same breathing conditions: their standard deviations are almost equal, while their averages show a difference of ~0.1 s.

Figures 5B,D show the probability distribution of the delivery time and of the gas supply ratio, respectively, but within the reference geometry that has been altered into a pathological scenario. In this scenario, 70% of the terminal airways, those farthest from the trachea, have ϕ_*i,j*_ set to 0.5 in order to mimic a situation in which 70% of the acini would have the inner diameter of their airways reduced by 50% due to swelling or an accumulation of liquid. Regarding the delivery time, this pathological scenario causes a slight decrease of its average, from 1.23 to 1.18 s, due to some acini not receiving fresh gas anymore (if *t*_del_*i,j*__ < 0 is calculated, it is set to zero) and a relative increase of its standard deviation of 38%. For the gas supply ratio, there is a strong relative increase of its standard deviation, of 83%. The results show that the pathological situation leads to a bimodal distribution of *t*_del_ and Ω, with a first peak corresponding to the blocked acini and a second one corresponding to the free ones. This is especially seen on the probability distribution of Ω (see Figure 5D): a first maximum is observed for a value of Ω significantly lower than 1, representing the 70% of the acini that are blocked and receive less gas, and a second maximum is observed for a value of Ω higher than 1, representing the 30% of the acini that are free and receive more gas than usual because of redirected gas from the neighboring blocked acini.

In conclusion, this analysis illustrates that our model is able to characterize the convective transport of a gaseous species, such as oxygen for instance, along the tracheobronchial tree and its delivery to the acini. This could allow to mimic situations in healthy lungs as well as in unhealthy lungs and gain new insights on gas (re)distribution in the distal end of the tracheobronchial tree.

#### 3.1.2. Air Flow in Lungs With COPD

COPD, or chronic obstructive pulmonary disease, is a lung condition characterized by progressive airway obstruction and airway inflammation, causing poor airflow and breathing difficulties (Decramer et al., 2012; Vestbo et al., 2013). The chronic airway limitation is the result of the breakdown of lung tissue (known as emphysema), and of small airways (*D*_*i,j*_ < 2 mm) disease (known as obstructive bronchiolitis) (Vestbo et al., 2013). In order to mimic this with our model, we can, regarding the obstructive bronchiolitis, locally strongly reduce (with β = 0.99, see Equation (19) in the next section) the diameter of a portion of the small airways (*D*_*i,j*_ < 2 mm) of the tracheobronchial tree and, concerning emphysema, “block” some acini by setting ϕ_*i,j*_ = 0.01 for certain terminal airways, given that emphysema is essentially affecting the intra-acinar region.

**Figure 6:**
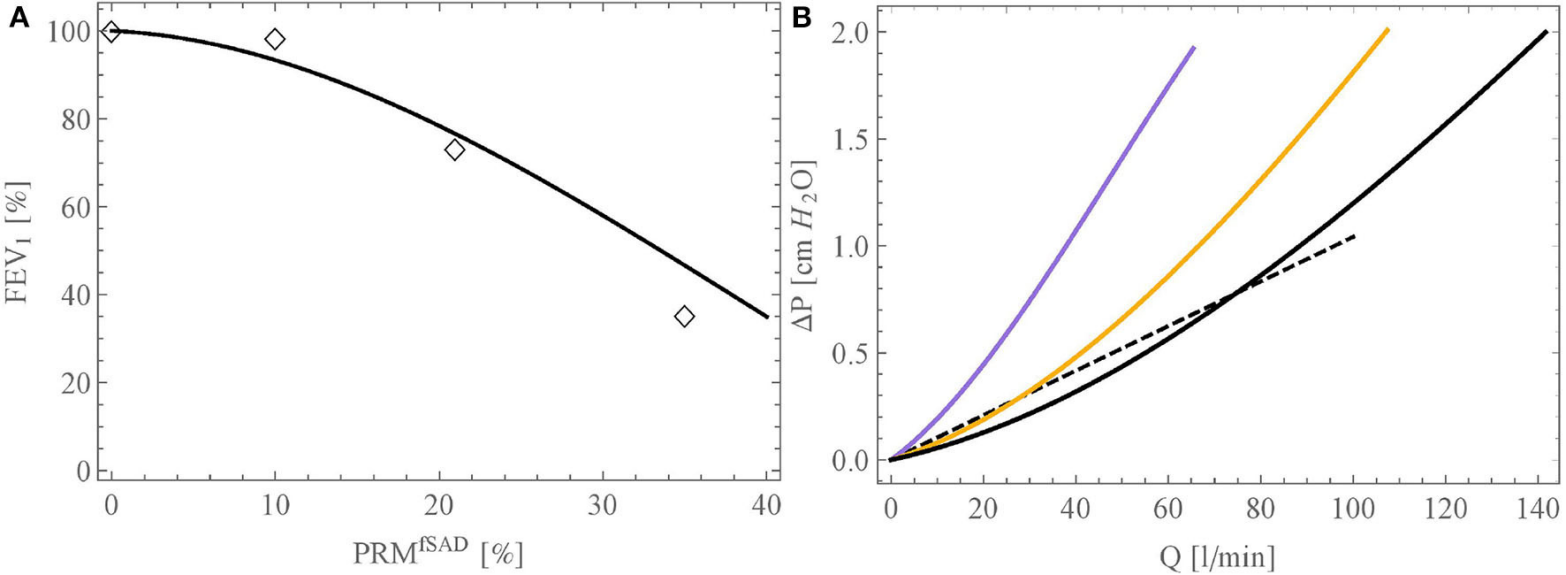
**(A)** Link between FEV_1_ (in % of its reference value in healthy subjects) and PRM^SAD^ obtained with our model (black curve). The open diamonds represent the experimental data of Criner et al. (2019). **(B)** Relation between inspiration flow rate and pressure drop in the bronchial tree, calculated with the model, for three situations: healthy lungs (black curve), lungs with PRM^fSAD^ = 21% (orange curve), and lungs with PRM^fSAD^ = 35% (purple curve). The dashed curve presents the linear relation of Ferris et al. (1964).

First, we compare our model against the data of Criner et al. (2019). These authors analyzed data in 1,846 individuals with ≥10 pack-years smoking history and for whom forced expiratory volume in 1 s (FEV_1_) and CT imaging data were available and obtained on the same day. Based on the CT data, two factors, characterizing the extent of the COPD, were determined: the quantification of the areas of emphysema (PRM^Emph^, expressed in %) and of the areas of small airway disease (PRM^fSAD^, expressed in %). In their article, the authors present a table giving, for different classes of patients, the average values of FEV_1_, PRM^fSAD^ and PRM^Emph^. Using this, the link obtained between FEV_1_ (in % of its reference value in healthy subjects) and PRM^fSAD^ is shown in Figure 6A (empty diamonds). To analyze this with our model, we first interpolated the authors data to yield a continuous link between PRM^fSAD^ and PRM^Emph^. Then, we performed several simulations, for varying values of PRM^fSAD^, imposing an alveolar overpressure of 250 Pascals (because this value leads with our model, using the reference geometry, to an expiration flow rate of 2.78 l/s, corresponding to the average FEV_1_ of healthy subjects mentioned by Criner et al., 2019). For each simulation, we randomly selected a percentage PRM^fSAD^ of the small airways (*D*_*i,j*_ < 2 mm) and reduced their lumen area by 99% (β = 0.99). Additionally, we also selected the related percentage PRM^Emph^ of the terminal airways and blocked their subsequent acini with ϕ_*i,j*_ = 0.01. The resulting link obtained between FEV_1_ (in % of its reference value in healthy subjects) and PRM^fSAD^ is shown in Figure 6A (continuous curve). A good qualitative agreement is obtained with the clinical data, showing that our model is able to capture the complex dynamics of air flow in lungs with COPD. The non-linear nature of the relation between FEV_1_ and PRM^fSAD^ can be explained by the fact that, in a healthy subject, it is primarily the proximal airways that are responsible for the resistance to the flow in the lungs, due to the undeveloped character of the flow within them. Consequently, a marked damage to the distal airways is required for FEV_1_ to decrease significantly.

Second, Figure 6B presents the relation between inspiration flow rate and pressure drop in the bronchial tree, calculated with the model, for three situations: healthy lungs (black curve), lungs with PRM^fSAD^ = 21% (orange curve), and lungs with PRM^fSAD^ = 35% (purple curve). The linear relation presented by Ferris et al. (1964) is also given on the figure (dashed curve). For a healthy subject, our model predicts lower pressure drops than those given by Ferris et al., up to a flow rate of about 70 l/min, and then higher pressure drops. This behavior is quite similar to the results obtained by Pedley et al. (1970b). The flow-pressure drop relation predicted by our model is non-linear, due to the importance of the resistance to the flow in the first generations, which is flow-dependent due to the introduction of the factor *Z*_*i,j*_. Finally, this figure shows that the model allows characterizing the respiratory limitations imposed by COPD in a patient. For example, it is observed that a pressure drop four times larger must be created in a severely affected patient, compared to a healthy person, for breathing at rest (around of 15 l/min). We also see the very significant limitation that COPD brings for breathing during an effort, with inspired volumes being up to three times less than for a healthy subject, at a constant depression created by the diaphragm.

Finally, it should be noted that the simulation results presented in Figure 6 are those obtained for a given geometry and alteration. Depending on the inherent local anatomic variability (characterized by σ_*i*_) and the location of the small airways affected by obstructive bronchiolitis, the results may vary. However, it has been verified that this variability does not impact significantly the calculated curves.

### 3.2. NO Exchange and Transport

In this section, results obtained with the model are put in parallel with existing experimental or modeling works regarding the dynamics of NO in the lungs. Notably, they are used to shed new light on this dynamics in unhealthy lungs.

#### 3.2.1. NO Exchange Between the Bronchial Wall and the Lumen

In the airway (*i, j*) of the tracheobronchial tree, the total amount of NO arriving per unit of time from the airway wall into the lumen, *F*_*i,j*_, can be calculated with Equations (11) and (12). This equation shows, logically, that this flux is modified when the geometrical parameters defining the airway wall (lumen diameter and layer thicknesses) are altered. In the case of a lung pathology, such as asthma, CF, or COPD, the inner diameter of an airway can experience changes due to several causes. In this work, two types of airway alterations are considered: a contraction of the smooth muscle of the airway (the tissue layer in Figure 6) and an accumulation of liquid in the airway (mucus for instance in the case of CF). A contraction of the smooth muscle of the airway (*i, j*) causes a change in *D*_*i,j*_ as well as in δ_μ_*i,j*__, δ_*E*_*i,j*__, and δ_*M*_*i,j*__, whereas an accumulation of liquid only affects *D*_*i,j*_ and δ_μ_*i,j*__. The way these different parameters are adjusted in the case of an airway alteration is the same as the one applied by Karamaoun et al. (2016). In any case, the extent of the decrease of the lumen diameter is defined by a lumen reduction factor, β_*i,j*_:

(19) $$\begin{array}{l} \beta_{i,j}=1-{\left(\frac{D_{i,j}^{\text{altered}}}{D_{i,j}}\right)}^{2} \end{array}$$

with $D_{i,j}^{\text{altered}}$ the diameter of the lumen of the airway (*i, j*) after its alteration.

**Figure 7:**
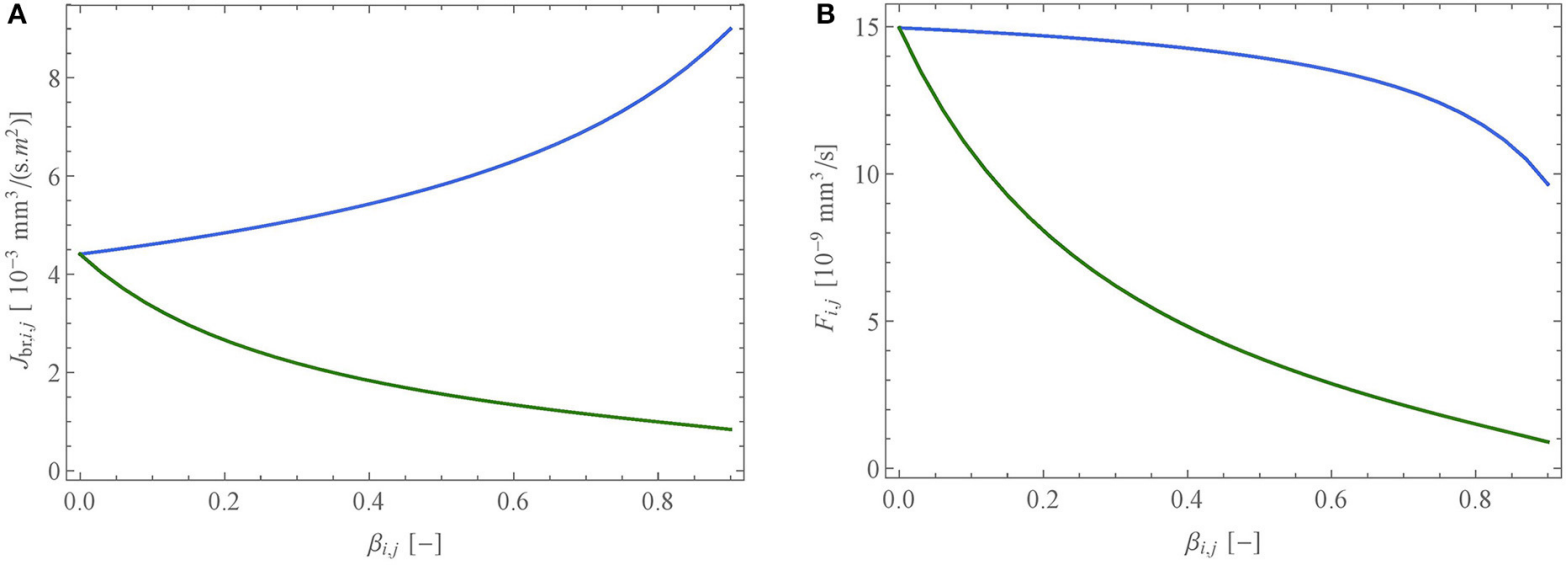
Impact of the reduction of the lumen of an airway (*i, j*) (with an initial diameter of 600 μm), expressed through the lumen reduction factor, β_*i,j*_, due to a muscle contraction (blue) or an accumulation of liquid (green), onto **(A)** the NO transfer flux density between the wall of an airway and the lumen, *J*_br,*i,j*_, and **(B)** the total amount of NO delivered per unit of time in the airway, *F*_*i,j*_. The quantities are calculated using the ideal gas law at atmospheric pressure and at 37°C.

Figures 7A,B show *J*_*br, i, j*_ and *F*_*i,j*_ as functions of β_*i,j*_, for a muscle contraction (blue curve) or an accumulation of liquid (green curve), for an airway with an initial lumen diameter of 600 μm and for a constant value of the NO volumetric production rate in the epithelium, *P*.

Figure 7A shows that *J*_br,*i,j*_, the NO transfer flux density between the wall of the airway and the lumen, depends on β_*i,j*_ in a very different way, depending on whether the reduction of the lumen is due to a contraction of the muscle or to an accumulation of liquid. Indeed, *J*_br,*i,j*_ is increased in the case of a muscle contraction and decreased in the case of an accumulation of liquid. The reduction in the case of an accumulation of liquid is obvious as the latter acts as a barrier between the site of NO production, the epithelium, and the lumen, everything else being constant. The increase of the flux density in the case of the muscle contraction is more subtle to understand. To estimate the new values of the thicknesses of the liquid, epithelium and muscle layers following a contraction of the muscle, we assume that their volumes are conserved. As a result, their thicknesses are increased. Consequently, the residence time of a NO molecule in the epithelium increases (as it is transported by diffusion, this residence time is proportional to the square of the thickness of the epithelium). As the volumetric production rate of NO in the epithelium is assumed constant, it results in an increase of the concentration of NO in the epithelium that overwhelms the thickening of the liquid layer, yielding an increased flux density.

On the contrary, Figure 7B shows that the NO transfer flux between the wall of the airway and the lumen, *F*_*i,j*_, decreases regardless of the scenario. In the case of an accumulation of liquid, this is obvious, since the flux density and the exchange surface between the airway wall and the lumen both decrease. In the case of a contraction of the muscle, the observed decrease of *F*_*i,j*_ implies that the exchange surface decreases more than the flux density increases.

As mentioned previously, in asthma patients, airway inflammation is traditionally monitored by measuring the molar fraction of NO in the exhaled air (F_E_NO) (Abba, 2009). This is easily understood given that *F*_*i,j*_ is proportional to the volumetric rate of NO production in the bronchial epithelium *P* (see Equations 11 and 12). However, the results presented in Figure 7B show that smooth muscle constriction, another feature of asthma, causes a decrease in *F*_*i,j*_, which could very well mask an increase in *P*. Such a compensation has actually been observed in the clinical study of Haccuria et al. (2014), using a clinical protocol triggering inflammation and constriction at different times in asthma patients. This shows that F_E_NO measurements should be analyzed with caution in certain asthma situations.

In CF patients, it has been reported that the F_E_NO is decreased when compared to a healthy population (Grasemann et al., 1997; Ojoo et al., 2005; Abba, 2009), despite CF being an inflammatory disease. Several elements have been proposed to explain this: an alteration of the transport of NO from the epithelium to the lumen, the removal of NO by reaction with reactive oxygen species in the inflamed environment and the failure of up regulation of epithelial iNOS in chronic suppurative conditions (Gemicioglu et al., 2014). The results presented in Figure 6B show that *F*_*i,j*_, and thus the F_E_NO, is strongly impacted by an accumulation of liquid in the airways. For example, these results show that even if, as an inflammatory response, the volumetric production rate of NO in the bronchial epithelium *P* is doubled (figure chosen by a parallelism with asthma, as it is commonly reported in asthma patients that the F_*E*_NO value is doubled when compared to a healthy population [1, 12]), a simultaneous reduction of the lumen area of 50% (i.e., β = 0.5) due to a sole accumulation of mucus, characteristic of CF, would yield a resulting flux *F*_*i,j*_ decreased by a factor 2 when compared to the base state (no inflammation and no liquid accumulation). This shows that the sole alteration of the transport of NO from its site of production to the lumen, by the accumulation of mucus, could explain the reduced F_E_NO observed in CF patients.

#### 3.2.2. NO Transport in Healthy Lungs

When a molecule of NO diffuses through the wall of an airway and reaches the lumen, the respiratory conditions and the location within the lungs determine whether it will be transported toward the mouth or toward the alveoli. If the Peclet number in the airway (*i, j*) is around 1, there is a fair competition between axial gas transport by convection and diffusion. During an expiration, this implies that a NO molecule can either be transported toward the mouth by convection or be dragged by diffusion into the alveolar space, where the NO concentration is low due to its rapid consumption by the blood. This diffusive transport to the alveoli, opposite to the expiratory flow direction, is called the back-diffusion (Verbanck et al., 2008). During a clinical measurement of the molar fraction of NO in the exhaled air, the contribution of the nasal cavity to the F_E_NO signal is minimized through the use of an expiratory resistance closing the palate. Therefore, the F_E_NO at the trachea can be assumed to be equal to the F_E_NO measured at the mouth (American Thoracic Society and European Respiratory Society, 2005).

**Figure 8:**
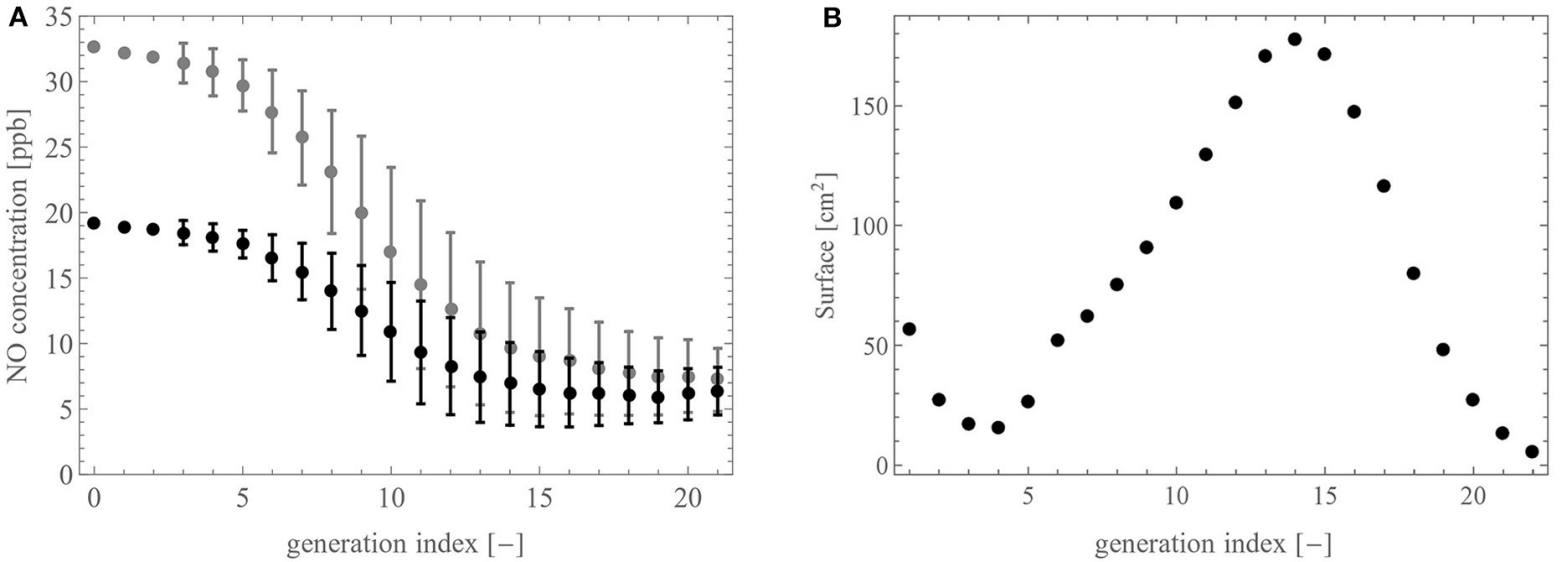
**(A)** NO concentration profile in the tracheobronchial tree at 25 ml/s (gray) and at 50 ml/s (black). **(B)** Total exchange area between the walls of the airways and the lumen in a generation, as a function of the generation index.

Figure 8A shows the NO concentration profile in the lungs calculated with the model, after a slow expiration at 25 ml/s (gray) and 50 ml/s (black). More precisely, we present the average and the standard deviation of the NO concentration at the distal end of the airways of a generation, as functions of the generation index (0 being the top of the trachea). By looking at the NO concentration profiles from the distal region of the lungs to the trachea (i.e., by following the gas flow), we see that they are first relatively flat (generation 22 until about generation 15), then they rise significantly in the generations 14 to 5, before being flat again over the proximal generations. This is easily explained with regard to the distribution of the terminal airways (see Figure 4A), mostly located in generations 15–17, which act as NO pumps via the back-diffusion, and the distribution of the exchange surface between the bronchial wall and the lumen (see Figure 8B), which is particularly important in generations 10 to 17. Therefore, when gas flows from the distal region of the lungs to the trachea, the supply of NO and its “pumping” by the acini initially compensate for each other, resulting in this almost flat profile in generations 22 to 15. Then, beyond generation 15 (going toward the mouth), there are less terminal airways but there still is a large exchange surface between the bronchial walls and the lumen. This results in this significant increase in the NO concentration from generation 15 to generation 5. Then, during its way through the proximal airways, the gas hardly receives any more NO due to the small exchange surface. This results in this flat profile over generations 5 to 1.

**Figure 9:**
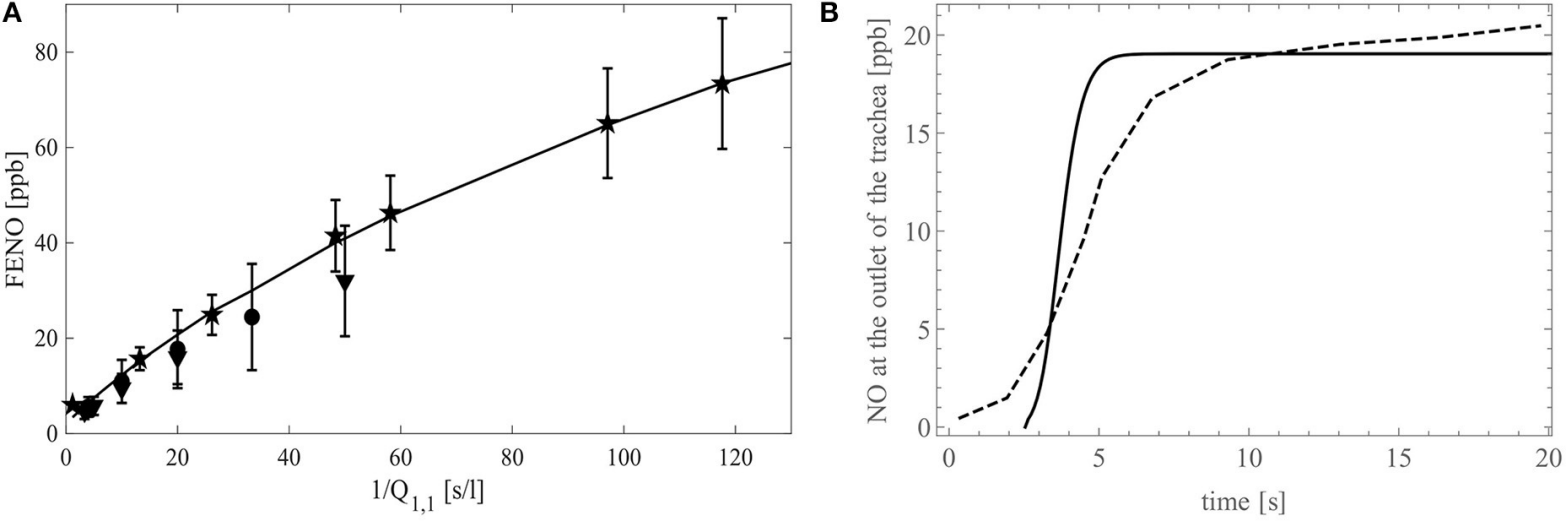
**(A)** F_E_NO (in ppb) as a function of the inverse of the flow rate in the trachea, 1/*Q*_1,1_ (in s/l). The stars represent the experimental data of Silkoff et al. (2000), the circles are the experimental data of Caspersen et al. (2011) and the upside down triangles are the experimental data of Malinovschi (2008). The symbols denote mean values and whiskers standard deviations. The simulation results obtained with our model are represented with the black full line. **(B)** F_E_NO (in ppb) as a function of time during a 20 s expiration at a flow rate of 50 ml/s. The dashed line shows the experimental data on a single healthy patient by Kerckx and Van Muylem (2009) and the simulation results obtained with our model are represented with the black full line.

In Figure 9A, F_E_NO is presented as a function of 1/*Q*_1,1_. The continuous line gives the modeling results, while the clinical data from the studies of Silkoff et al. (2000), Caspersen et al. (2011), and Malinovschi (2008) are given by the stars, circles and upside down triangles, respectively (the symbols denote mean values and whiskers standard deviations). A good agreement between the simulations results and the experimental data is observed. The modeling and the experimental results both show that the slope of the curve F_E_NO = *f*(1/*Q*_1,1_) decreases when 1/*Q*_1,1_ increases. This decrease highlights the impact of the back-diffusion on the F_E_NO. Indeed, for 1/*Q*_1,1_→ 0, the Peclet number in the entire tracheobronchial tree becomes way larger than 1, indicating that, regarding the axial transport, convection prevails against diffusion. Consequently, there is no back-diffusion and, given that *J*_br,*i,j*_ is independent of (*i, j*) in a healthy individual, F_E_NO ∝*S*_*t*_/*Q*_1,1_, with *S*_*t*_ the total area of the exchange surface between the bronchial walls and the lumen. As 1/*Q*_1,1_increases, the Peclet number in the distal airways decreases to values around or lower than 1. This implies a rise of the influence of the back-diffusion in these airways, causing some NO to be pumped toward the acini. As a result, F_E_NO increases less rapidly when 1/*Q*_1,1_increases.

Figure 9B presents the time evolution of the molar fraction of NO at the outlet of the trachea, during a slow expiration of 20 s at a flow rate of 50 ml/s. The dashed line represents experimental results on a single healthy patient obtained by Kerckx and Van Muylem (2009), whereas the full line represents the simulation results obtained with our model, using the numerical scheme to solve the time-dependent system (see Appendix 4). The comparison between these two graphs show that the model is in good qualitative agreement with the data. Although the data contains a small damping due to the time constant of the gas analyzer, the model calculates the sudden concentration rise too soon. This shows a limitation of general models of the lungs when they are compared to data of a single patient.

#### 3.2.3. NO Transport in Unhealthy Lungs

In this section, the F_E_NO is defined as the molar fraction of NO at the top of the trachea (this position is referred to by the generation index 0), at the end of a 10 s expiration with a flow rate of 50 ml/s.

Perez-Bogerd et al. (2019) identified a peripheral airway obstruction reversibility in COPD patients through F_E_NO measurements. They carried out a clinical study on 61 stable COPD patients and detected 3 different patterns of F_E_NO response to β_2_-agonists depending on the extend of the dilation process in the lungs. The first pattern is a F_E_NO decrease, possibly related to the relief of an intra-acinar airway obstruction. The second and third pattern are an increased F_E_NO or unchanged F_E_NO, most likely linked to a more proximal heterogeneous dilation. Similarly, Michils et al. (2016) highlighted the same patterns on F_E_NO response to β_2_-agonists during a clinical study on 68 asthmatic patients. They suggested a F_E_NO increase likely to be associated with a predominant dilation up to the pre-acinar airways and a F_E_NO stability to occur when the obstruction relief reaches predominantly the central airways. Karamaoun (2017) evaluated the use of the F_E_NO measurement in monitoring chest physiotherapy in CF patients. Their results showed that the F_E_NO response after chest physiotherapy was either increased, decreased or remained constant. Similarly to the results of Perez-Bogerd et al. (2019) and Michils et al. (2016), they suggested that the change in the F_E_NO is linked to the location in the tracheobronchial tree where mucus has been displaced during the physiotherapy session.

**Figure 10:**
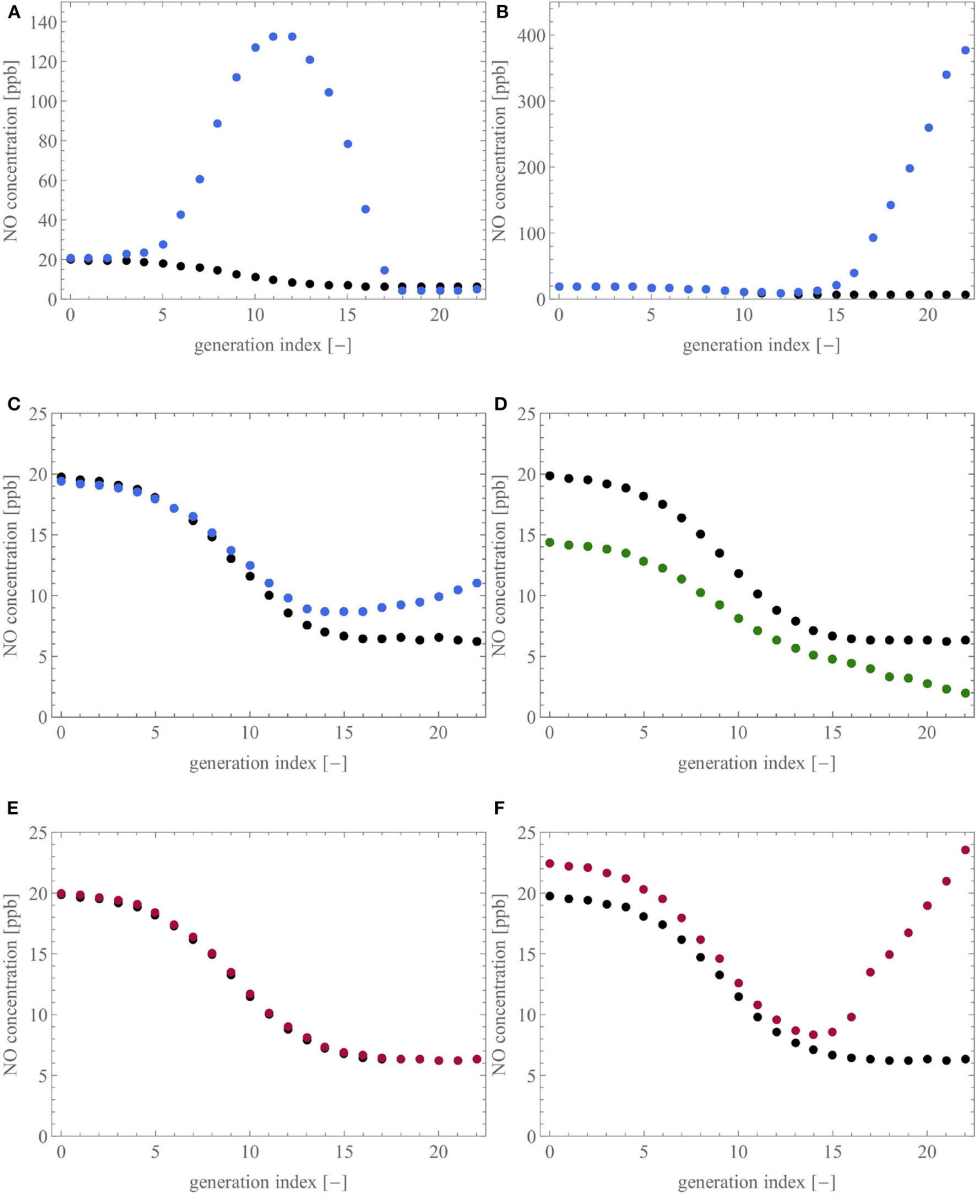
Influence of six different scenarios on the NO concentration profile in the lungs. For each subfigure, the healthy baseline, given in black, is obtained by performing expiration at 50 ml/s on the reference geometry. **(A,B)**: ϕ_*i,j*_ is set to 0.1, considering **(A)** 50% of the terminal airways, the ones closest to the trachea, or **(B)** the ones farthest from the trachea. **(C,D)**: 50% of the terminal airways and their three parents have their diameter reduced by 65%, by muscle contraction **(C)** or by an accumulation of liquid **(D)**. **(E,F)**: 50% of the terminal airways, considering **(E)** the ones closest to the trachea, have a value of γ_*i,j*_ reduced to 0.7 or **(F)** the ones farthest from the trachea, have a value of γ_*i,j*_ reduced to 10^−6^.

To analyse these features with our model, Figure 10 shows the influence of six different scenarios that could be encountered in unhealthy lungs on the NO concentration profile within these lungs, at the end of a slow expiration at 50 ml/s. Each profile is presented as the average NO concentration at the distal end of the airways of a generation, as a function of the generation index (the top of the trachea is generation 0). For each subfigure, the healthy baseline, given in black, is obtained using the reference geometry (see Table 1), and $\phi_{i,j}=\gamma_{i,j}=1,\forall \left(i,j\right)\in T$.

Figures 10A,B present two scenarios in which the parameter ϕ_*i,j*_ is set to 0.1 for 50% of the terminal airways to mimic a situation in which 50% of the acini have the inner diameter of their airways decreased by 90%. In Figure 10A, the acini closest to the trachea are considered while, in Figure 10B, the acini farthest from the trachea are considered. In accordance with the different clinical studies (Verbanck et al., 2008; Michils et al., 2016; Karamaoun, 2017; Perez-Bogerd et al., 2019), we observe that a reduction of the diameter of the airways at the intra-acinar level causes a strong increase of the NO concentration profile in the tracheobronchial tree and a slight increase of the F_E_NO (F_E_NO increase of 5.1% in Figure 10A and 2.7% in Figure 10B). Additionally, in Figure 10A, the NO concentration profile along the tracheobronchial tree has a local maximum, located between the average index of the considered terminal airways (generation 14) and the trachea. In both situations, the important accumulation of NO in the lungs is explained by the fact that the blocked acini have a reduced ability to attract the NO by diffusion. Moreover, the flow of air in the terminal airways upstream from the blocked acini is strongly reduced, due to the increase of their peripheral resistance, contributing also to the accumulation of NO in the distal region of the tracheobronchial tree.

Figures 10C,D present two scenarios where half of the terminal airways, those farthest from the trachea, and their three generations of parents have their diameter reduced by 65%. This reduction is obtained by muscle contraction (Figure 10C) or by an accumulation of liquid (Figure 10D). As discussed previously, the amount of NO delivered per unit of time in an airway is decreased in the case of muscle constriction or liquid accumulation (see Figure 7). Furthermore, the pre-acinar airways are the major production site for NO (see Figure 8). Consequently, the scenarios presented in Figures 10C,D logically lead to a decrease of the F_E_NO. The relative decrease of the F_E_NO is of 2.0% for the constricted case and of 27.7% for the case with the accumulation of liquid. The difference between the two scenarios (muscle contraction or liquid accumulation) can be related to the difference in response of *F*_*i,j*_ to a diameter reduction caused by muscle contraction or liquid accumulation, as discussed in the section 3.2.1. This relation between a decrease of the airway caliber in the pre-acinar airways and a decrease of the F_E_NO is in accordance with the experimental studies mentioned earlier (Verbanck et al., 2008; Michils et al., 2016; Perez-Bogerd et al., 2019).

Figures 10E,F present two scenarios where 50% of the terminal airways (those closest to the trachea in the case of Figure 10E and those farthest from the trachea in the case of Figure 10F) have a reduced value of γ_*i,j*_ (0.7 for Figure 10E and 10^−6^ for Figure 10F), to analyze a situation in which 50% of the acini have a reduced consumption of NO. In Figure 10E, almost no difference is observed between the healthy and the pathological situation (F_E_NO increase of 0.7%). This can be explained by the fact that NO has a very high affinity for the blood. Consequently, even if some acini are under-perfused, they still, to some extent, capture NO in the same way (due to the high value of *k*_alv_). However, this is only true for limited sub-perfusion because, in the case of a severe sub-perfusion (see Figure 10F, where $\gamma_{i,j}=10^{-6}$), the reduced consumption of NO by many acini leads to a marked increase of the NO concentration profile and, therefore, of the F_E_NO (increase of 13.5%).

In conclusion, the use of our model shows that changes in airway caliber, due to smooth muscle contraction or liquid accumulation, and perfusion defects cause changes in the NO concentration profile along the tracheobronchial tree. The model is able to put forward different types of F_E_NO changes, i.e., an increase of the F_E_NO, compared to the healthy state, when the airway caliber decrease takes place in the intra-acinar airways, and a decrease of the F_E_NO, when the airway caliber decrease takes place in the pre-acinar airways. These different patterns of F_E_NO changes are in accordance with what has been previously shown in clinical studies (Verbanck et al., 2008; Michils et al., 2016; Karamaoun, 2017; Perez-Bogerd et al., 2019). Additionally, the model demonstrates that the same type of change in the F_E_NO (increase or decrease) can be caused by different pathological situations and that small variations of F_E_NO may hide important alterations taking place in the distal region of the lungs. In this way, the model may contribute to the understanding of the role of NO as a marker for lung disease, such as asthma, CF, or COPD. It is important to stress that the phenomena highlighted in Figure 10 could not have been unraveled by a mathematical model based on a homogeneous description of the lung geometry. Indeed, as mentioned in the introduction, with such a homogeneous representation of the lung geometry, the airways are not individually represented in the model, and therefore, cannot be individually altered.

### 3.3. Model Limitations

Despite being quite general, the model has several limitations. First, it is well-known that the flow in the first generations of the lungs can be turbulent, especially at high inspiration/expiration flow rates. This feature is not included in the model, but it has limited impact on the global resistance to the flow of the lungs, which is on the other hand strongly influenced by the undeveloped character of the flow in the proximal airways (and this feature is included in the model through the parameter *Z*_*i,j*_) (Pedley et al., 1970a,b). Turbulence also enhances the dispersion, this is classically modeled by introducing a turbulent diffusion coefficient in the transport equations. This is not done in the model but it would have a very limited impact on the results, according to the very high Peclet numbers in the first generations, making this turbulent dispersion having a negligible influence on the transport.

Second, the model considers constant dimensions of the airways in the tracheobronchial tree. This might hide some dilution effect of the exchanged species, especially at low tidal volume.

Another limitation of our model is the simplified representation of the acini (1D quasi-steady approach, constant dimensions…), compared to previous works (Felici et al., 2005; Hofemeier et al., 2016). But it should be noted that, through the “linking function” *f*^±^ (see Equation 14), this simplified representation makes it possible to easily consider the acini in the complete model of the lungs and to assign specific parameters to each of them. Concerning the alterations that can be made to the acini in the model, the one concerning a modification of the perfusion through the parameter γ is quite realistic, in the sense that it amounts to reducing the coefficient *k*_alv_ of the considered acini. The other possible alteration, introduced via the parameter ϕ to mimic swelling or liquid accumulation, is less realistic as it simply consists in increasing the resistance to the flow of the considered acini and reducing the diffusive flux at their entrance.

Finally, as mentioned in the introduction, the model is based on an idealized representation of the geometry of the lungs, a geometry which is nevertheless person-dependent. Therefore, the purpose of a model, such as the one developed in this paper is above all to qualitatively reproduce experimental data and to highlight interesting phenomena, in particular to shed new light on clinical data/experimental observations.

## 4. Conclusion

In this work, a new mathematical model of the transport and exchange of a gas species in the lungs is presented and used. Compared to previous models, its main new feature is the ability to generate a fully tunable lung geometry of the tracheobronchial tree, using a branching asymmetry. Within this geometry, the model includes a method to calculate the gas flow distribution, the evaluation of the exchange fluxes of a gas species between the tissues and the lumen, and the computation of the concentration profile of the exchanged species in the lumen of the tracheobronchial tree. Moreover, the model has the flexibility to reproduce different scenarios encountered in lung pathologies, including constrictions of the airways, accumulation of liquid in the airways and perfusion defects in the alveolar region. The model allows to recreate stationary or time-dependent physiological situations of gas transport and exchange in the tracheobronchial tree. It has been checked that the model gives results coherent with experimental information available in the literature.

The simulation results show how, under specific respiratory conditions, the model can give new insights into the flow of gas in the lungs and how this is affected in altered lungs, for instance when affected by COPD. Simulation results dedicated to NO, mimicking situations encountered in different pathologies, such as asthma, CF, or COPD, highlight the impact of the disease onto the air flow distribution, the NO exchange flux between the tissues and the lumen and onto the NO concentration profile in the lungs, as well as the variety of possible F_E_NO responses.

In conclusion, the results show how the new model could serve for clinical purposes as a tool to unravel the dynamics of gas transport and exchange in unhealthy lungs, in particular in their distal region.

## Data Availability Statement

The original contributions presented in the study are included in the article/Supplementary Material, further inquiries can be directed to the corresponding author/s.

## Author Contributions

AB, BH, AN, and AV designed the main characteristics of this article. AB and BH wrote the article. All authors contributed to the article and approved the submitted version.

## Conflict of Interest

The authors declare that the research was conducted in the absence of any commercial or financial relationships that could be construed as a potential conflict of interest.

## Figures and Tables

**Table:** 

*Roman symbols*
A	Set of all the airways in the tracheobronchial tree
B	Set of all the intermediate airways in the tracheobronchial tree
*C*	Concentration, mol/m^3^
CF	Cystic Fibrosis
COPD	Chronic Obstructive Pulmonary Disorder
*d*_alv_	Inner diameter of the alveoli, m
*D*	Inner diameter of the airway (i.e., diameter of the lumen), m
D	Diffusion coefficient, m^2^/s
*F*	NO exchange flux, mol/s
F_E_NO	Fraction of Exhaled Nitric Oxide, ppb
*h*	Ratio between the diameter of the airway and the diameter of its parent airway, –
Ha	Hatta number, –
*J*	NO exchange flux density, mol/(m^2^.s)
*k*_alv_	Mass transfer coefficient in the alveoli, m/s
*k*	Kinetic constant, s^−1^
*L*	Length of the airway, m
*n*_alv,*m*_	Number of alveoli in generation *m* of an acinus, –
NO	Nitric Oxide
Pe	Peclet number, –
*P*	Volumetric NO production rate in the epithelial layer, mol/(m^3^.s)
P	Pressure in the alveolar sacs, Pa
*Q*	Gas flow rate, m^3^/s
*r*	Radial coordinate in the airway, m
*R*_*L*_	Distance between the center of an airway and the liquid-lumen interface, m
Re	Reynolds number, –
R	Resistance to the flow, Pa.s
*t*	Time, s
T	Set of all the terminal airways in the tracheobronchial tree
*V*	Average axial velocity in the airway, m/s
*X*	Normal distribution
*z*	Axial coordinate in the airway, oriented in the direction of the alveolar region, m
*z*′	Axial coordinate in the airway, oriented in the direction of the flow, m
*Greek symbols*
α	Ratio between the length and the diameter of the airway, –
β	lumen reduction factor, –
γ	Perfusion coefficient, –
δ	Tissue thickness, m
ν	Kinematic viscosity of the gas, m^2^/s
ρ	Density of the gas, kg/m^3^
σ	Standard deviation
ϕ	Parameter to simulate swelling or liquid accumulation in the acini downstream of a terminal airway, –
χ	Asymmetry parameter, –
*Subscripts*
0	At rest
*a*	Acinus
alv	Alveolus
airw	Airway
br	Tracheobronchial tree
*E*	Epithelium
eq	Equilibrium
*g*	Gas phase
*i*	Generation number
*j*	Airway number in a generation
*L*	Lumen
lim	Limit value
*M*	Tissue
*m*	Generation number in an acinus
μ	Liquid
*t*	Tissue
term	Terminal
total	Total (sum over the set of all the airways in the tracheobronchial tree, A)
*Superscripts*
+	Inspiration
−	Expiration
sub	Subtree
