# Supplementary material for: Modeling of the Transport and Exchange of a Gas Species in Lungs With an Asymmetric Branching Pattern. Application to Nitric Oxide
Source: Front Physiol. 2020 Dec 10;11:570015. doi: 10.3389/fphys.2020.570015 (PMC7758446; doi:10.3389/fphys.2020.570015)
Supplement: Supplementary file 2 [file Data_Sheet_2.pdf]

APPENDIX 1 - CALCULATION OF THE GAS FLOW DISTRIBUTION IN THE TRACHEOBRONCHIAL TREE

In this Appendix, we describe how the model calculates the gas flow rate, $Q_{i,j}$, and the average axial gas velocity, $V_{i,j} = \frac{4Q_{i,j}}{\pi D_{i,j}^2}$, in each airway $(i,j) \in \mathcal{A}$. To establish the system of equations to calculate these parameters during inspiration or expiration, two parameters are introduced hereto.

The first parameter is the resistance to the flow in the airway (i,j), $\mathcal{R}_{i,j}$. It is defined as the Poiseuille resistance to the flow in this airway, $\mathcal{R}_{i,j}^{P}$, multiplied by a factor, $Z_{i,j}$ [4, 5]:

$$\mathcal{R}_{i,j} = Z_{i,j}\mathcal{R}_{i,j}^{P} = Z_{i,j}\,\frac{128\rho\nu L_{i,j}}{\pi D_{i,j}^4} \tag{1.1}$$

$\mathcal{R}_{i,j}^{P}$ is the minimal resistance to the flow of an incompressible viscous fluid through a straight cylindrical pipe of length $L_{\text{i,j}}$ and diameter $D_{\text{i,j}}$, with ρ the density of the gas and ν its kinematic viscosity. It corresponds to a Poiseuille flow (i.e. a parabolic velocity profile) that is developed throughout the entire length of the airway. However, depending on the breathing conditions and on the airway size, the velocity profile in an airway is not a fully developed Poiseuille flow and, consequently, the dissipated energy (and hence the resistance $\mathcal{R}_{i,j}$) is not minimal, due to the increased velocity gradients at the wall of the airway. Therefore, the factor $Z_{i,j}$ is calculated in our model as the ratio of the real energy dissipated in the airway (i,j) to the minimal energy that could be dissipated in that airway:

$$Z_{i,j} = \frac{\frac{1}{L_{i,j}}\displaystyle\int_0^{L_{i,j}} \frac{V_{i,j}}{\sqrt{\nu\frac{z'}{V_{i,j}}}}\,dz'}{\frac{8\,V_{i,j}}{D_{i,j}}} = \frac{1}{4}\sqrt{\frac{\text{Re}_{i,j}}{\alpha_i}} \tag{1.2}$$

In Equation 1.2, the numerator is the average, along the airway, of the velocity gradient at the wall of the airway in a developing flow, where the thickness of the viscous boundary layer, $\sqrt{\nu z'/V_{i,j}}$, is expressed as a function of z', the axial coordinate in the airway, orientated in the direction of the flow and with $z' = 0$ at the entrance of the airway. The denominator is the velocity gradient at the wall of the airway if a Poiseuille flow was fully developed in the airway. Developing this ratio, $Z_{i,j}$ can be expressed as a function of the Reynolds number of the flow in the airway (i,j), $\text{Re}_{i,j} = D_{i,j}V_{i,j}/\nu$. If $Z_{i,j}$ is evaluated as being smaller than 1, it is set to 1. The expression of $Z_{i,j}$ obtained with this approach is, apart from a constant proportionality factor, equal to 1.31, the same as the one given by the well-known equation developed by Pedley et al. [4, 5]. Using either Equation 1.2 or Pedley's equation in our model does not lead to any significant difference in the results.

The second parameter is the subtree resistance, $\mathcal{R}_{i,j}^{\text{sub}}$, of the airway (i,j). It gives the resistance to the flow exhibited by the whole part of the lungs that is downstream of the airway (i,j). This resistance is calculated in two different ways, given in Equation 1.3, depending on whether the airway considered is intermediate (i.e. $(i,j) \in \mathcal{B}$) or terminal (i.e. $(i,j) \in \mathcal{T}$):

$$\mathcal{R}_{i,j}^{\text{sub}} = \begin{cases} \left(\frac{1}{\mathcal{R}_{i+1,2j}+\mathcal{R}_{i+1,2j}^{\text{sub}}} + \frac{1}{\mathcal{R}_{i+1,2j-1}+\mathcal{R}_{i+1,2j-1}^{\text{sub}}} \right)^{-1}, & \textbf{if } (i,j) \in \mathcal{B} \\ \frac{1}{\phi_{i,j}^3} \frac{1}{2} \sum_{m=1}^{7} \frac{1}{2^{m-1}} \frac{128\rho\nu \, L_a}{D_a} = \frac{1}{\phi_{i,j}^3} \frac{254}{2} \frac{\rho\nu L_a}{D_a}, & \textbf{if } (i,j) \in \mathcal{T} \end{cases} \tag{1.3}$$

For an intermediate airway, an electrical analogy is used to calculate its subtree resistance (first line of Equation 1.3), as illustrated in Figure 1.1. For a terminal airway, the subtree resistance is the resistance to the flow of the two acini, in parallel, located after this particular airway (second line of Equation 1.3). The resistance to the flow in an acinus is calculated assuming a Poiseuille flow in each of its generations, as the Reynolds number of the flow in an airway of an acinus is usually smaller than 1. When the lung geometry is generated by the model, the value of 1 is assigned to the parameter $\phi_{i,j}$ appearing in Equation 1.3. However, by using some of the "alteration functions" mentioned previously, the user of the model can later reduce its value to a number strictly between 0 and 1 for some terminal airways. This allows to mimic the effect of alterations in the acini downstream of these airways (swelling, accumulation of liquid...) on the resistance to the flow in these acini. $\phi_{i,j}$ is set to the power of three in the denominator on the right-hand side of Equation 1.3 (second line) so that $\phi_{i,j}$ represents a reduction factor of the linear dimensions of the lumen of the acini downstream of the airway (i,j).

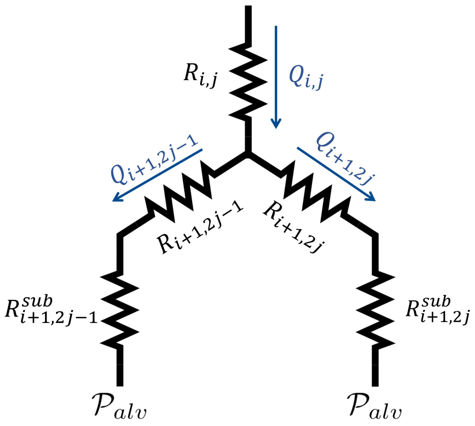

Figure 1.1. Electrical equivalence of a branching structure.

To couple the subtree resistances to the airway resistances and the flow rates, the electrical analogy is also used and Equations 1.4 and 1.5 are introduced, $\forall (i,j) \in \mathcal{B}$. Equation 1.5 relies on the assumption of a homogeneous alveolar pressure, $\mathcal{P}_{\text{alv}}$. The total tracheobronchial tree resistance, $\mathcal{R}_{\text{total}}$, is expressed in Equation 1.6. Finally, Equation 1.7, expressing the total pressure drop of the flow in the lungs, can also be written.

$$Q_{i,j} = Q_{i+1,2j} + Q_{i+1,2j-1} \tag{1.4}$$

$$Q_{i+1,2j}(\mathcal{R}_{i+1,2j} + \mathcal{R}_{i+1,2j}^{\text{sub}}) = Q_{i+1,2j-1}(\mathcal{R}_{i+1,2j-1} + \mathcal{R}_{i+1,2j-1}^{\text{sub}}) \tag{1.5}$$

$$\mathcal{R}_{\text{total}} = \mathcal{R}_{1,1} + \mathcal{R}^{\text{sub}}_{1,1} \quad (1.6)$$

$$-\mathcal{P}_{\text{alv}} = \mathcal{R}_{\text{total}}\, Q_{1,1} \quad (1.7)$$

Equations 1.1 to 1.7 allow, for a given value of $Q_{1,1}$ or $\mathcal{P}_{alv}$ (possibly time-dependent), calculating the gas flow rate and the resistance to the flow within each airway in the tracheobronchial tree. This system of equations is solved using an iterative procedure sketched in Figure 1.2.

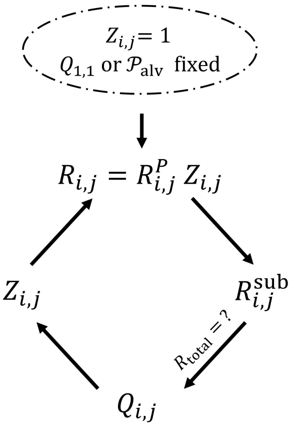

Figure 1.2. Representation of the iterative procedure to determine the flow rates $Q_{i,j}$ and the resistances $\mathcal{R}_{i,j}$ in the tracheobronchial tree.

This procedure starts with the hypothesis that all the airway resistances are equal to those of a Poiseuille flow (i.e. $Z_{i,j} = 1$). Next, all subtree resistances are calculated with Equation 1.3, starting from the terminal airways and then going up the tracheobronchial tree to the trachea. Subsequently, the flow rates are calculated, using Equations 1.4 and 1.5, starting from the top of the bronchial tree and going to the terminal airways. Then, the new values of $Z_{i,j}$ are calculated, using Equation 1.2, allowing to adjust the initial estimations of the airway resistances. This cycle is repeated until the difference between $\mathcal{R}_{\text{total}}$ and its value calculated in the previous cycle is smaller than 0.1%. It should be noted that the equations and methodology to calculate the gas flow distribution in the tracheobronchial tree are valid for an inspiration, as depicted in Figure 1.2, as well as for an expiration.

APPENDIX 2 - EXCHANGE OF NO BETWEEN THE BRONCHIAL WALL AND THE LUMEN OF AN AIRWAY

In this appendix, we describe how the representation of the phenomena taking place in the bronchial wall, described in section 2.3.1 of the article, are translated into equations, allowing to obtain an analytical expression of the NO exchange flux density between the wall of the airway (i, j) and its lumen, $J_{\text{br},i,j}$. First, a radial coordinate, r, is introduced together with the inner radius of the lumen, $R_{L_{i,j}}$ (see Figure 3). Then, different assumptions are made. Firstly, as the airway wall thickness is small compared to the airway length, only the radial diffusive transport of NO in the airway wall is considered. Secondly, we can evaluate that the NO concentration in the liquid at the level of the liquid-lumen interface (equal to HC_g if a local equilibrium is achieved at this interface, with H the Henry's coefficient of NO in water [1] and C_g the NO

concentration in the lumen) is way smaller than the NO concentration encountered in the epithelium layer (whose order of magnitude is $P(\delta_{\mu_{i,j}} + \delta_{E_{i,j}})^2/\mathcal{D}_t$), for common values of C_g in the lungs during a $\mathrm{F_E NO}$ test. Therefore, we assume that the NO concentration in the liquid layer, at its interface with the lumen, is zero. Thirdly, because of the high affinity of NO for the haemoglobin in the blood, we assume that the blood capillaries act as an infinite sink for NO and therefore that the NO concentration at the outer end of the tissue layer is zero. As a consequence of the second and third assumption, we can assume that the NO concentration profile in the wall of the airway is at stationary state, as the conditions at its boundary are time-independent (zero concentration). Using these assumptions, Equations 2.1 and 2.2, describing the production and the radial diffusive transport of NO in the wall of the airway, can be set. In these equations, $C_\mu(r)$, $C_E(r)$ and $C_M(r)$ are the NO concentrations at the radial position r in the liquid, epithelium and tissue layer, respectively.

$$
\begin{aligned}
&\frac{\mathcal{D}_t}{r}\frac{\partial}{\partial r}\left(r\frac{\partial C_\mu}{\partial r}\right) = 0 && \textbf{if} \quad R_{L_{i,j}} < r < R_{L_{i,j}} + \delta_{\mu_{i,j}} \\
&\frac{\mathcal{D}_t}{r}\frac{\partial}{\partial r}\left(r\frac{\partial C_E}{\partial r}\right) + P - k_t C_E = 0 && \textbf{if} \quad R_{L_{i,j}} + \delta_{\mu_{i,j}} < r < R_{L_{i,j}} + \delta_{\mu_{i,j}} + \delta_{E_{i,j}} \\
&\frac{\mathcal{D}_t}{r}\frac{\partial}{\partial r}\left(r\frac{\partial C_M}{\partial r}\right) - k_t C_M = 0 && \textbf{if} \quad R_{L_{i,j}} + \delta_{\mu_{i,j}} + \delta_{E_{i,j}} < r < R_{L_{i,j}} + \delta_{\mu_{i,j}} + \delta_{E_{i,j}} + \delta_{M_{i,j}}
\end{aligned} \tag{2.1}
$$

$$
\begin{aligned}
&C_\mu\big|_{r=R_{L_{i,j}}} = 0 && , C_M\big|_{r=R_{L_{i,j}}+\delta_{\mu_{i,j}}+\delta_{E_{i,j}}+\delta_{M_{i,j}}} = 0 \\
&C_\mu\big|_{r=R_{L_{i,j}}+\delta_{\mu_{i,j}}} = C_E\big|_{r=R_{L_{i,j}}+\delta_{\mu_{i,j}}} && , C_E\big|_{r=R_{L_{i,j}}+\delta_{\mu_{i,j}}+\delta_{E_{i,j}}} = C_M\big|_{r=R_{L_{i,j}}+\delta_{\mu_{i,j}}+\delta_{E_{i,j}}} \\
&\left.\frac{\partial C_\mu}{\partial r}\right|_{r=R_{L_{i,j}}+\delta_{\mu_{i,j}}} = \left.\frac{\partial C_E}{\partial r}\right|_{r=R_{L_{i,j}}+\delta_{\mu_{i,j}}} && , \left.\frac{\partial C_E}{\partial r}\right|_{r=R_{L_{i,j}}+\delta_{\mu_{i,j}}+\delta_{E_{i,j}}} = \left.\frac{\partial C_M}{\partial r}\right|_{r=R_{L_{i,j}}+\delta_{\mu_{i,j}}+\delta_{E_{i,j}}}
\end{aligned} \tag{2.2}
$$

The boundary conditions, given by Equations 2.2, include the zero concentration set in the liquid at the interface with the lumen and in the tissue at its interface with the blood capillaries, the continuity of the concentration and the conservation of the diffusive flux at the liquid-epithelium and epithelium-tissue interfaces.

The resolution of the system composed of Equations 2.1 and 2.2 yields an analytical expression for the transfer flux density of NO from the wall of the airway to the lumen, $J_{\mathrm{br},i,j}$. This expression is not presented here due to its prohibitive size. However, a simplified analytical expression can be derived. It is obtained from the full analytical solution through a Taylor expansion of the second order with respect to the consumption kinetic constant, k_t, and to the thicknesses of the layers of the wall of the airway. This yields:

$$
J_{\mathrm{br},i,j} = \mathcal{D}_t \left.\frac{\partial C_\mu}{\partial r}\right|_{r=R_L} \approx \frac{P\delta_{E_{i,j}}}{2} \frac{\delta_{E_{i,j}} + 2\delta_{M_{i,j}}}{\delta_{\mu_{i,j}} + \delta_{E_{i,j}} + \delta_{M_{i,j}} + \frac{\delta_{\mu_{i,j}}}{2}\mathrm{Ha}_{i,j}^2} \tag{2.3}
$$

with $\mathrm{Ha}_{i,j} = (\delta_{E_{i,j}} + \delta_{M_{i,j}})\sqrt{k_t/\mathcal{D}_t}$.

We have observed that the results obtained with the complete analytical solution for $J_{\mathrm{br},i,j}$ are not significantly different from those obtained with the approximate solution given by Equation 2.3.

APPENDIX 3 - TRANSPORT OF NO IN AN ACINUS

To describe the transport of NO within an acinus, two assumptions are made. First, a quasi-steady state is assumed because of the small characteristic times of NO diffusion in the airways composing the acinus, when compared to the inspiration (or expiration) time. Second, as verified by Paiva et al. [2, 3], we assume that only the axial diffusive transport in the lumen of the acinus has to be considered in the transport equations (i.e. radial homogeneity in the lumen is assumed). Consequently, Equations 3.1 and 3.2, similar to those used by Kamaraoun et al. [1], are written to describe the NO dynamics in an acinus with a concentration $C_a(t)$ at its proximal end (i.e. its inlet during inspiration or its outlet during expiration) and with a gas flow rate $Q_a(t)$ (defined as positive) entering or leaving the acinus:

$$\pm V_m \frac{\partial C_{g,m}(z,t)}{\partial z} = \frac{\pi D_a^2}{4} \mathcal{D}_g \frac{\partial^2 C_{g,m}}{\partial z^2} + \pi D_a J_{a,m} \qquad (m = 1, ..., 7) \tag{3.1}$$

$$\begin{aligned}
C_{g,1}\big|_{z=0,t} &= C_a(t) \\
C_{g,m}\big|_{z=L_a,t} &= C_{g,m+1}\big|_{z=0,t} \; (m = 1, ..., 6) \\
\left.\frac{\partial C_{g,m}}{\partial z}\right|_{z=L_a,t} &= 2 \left.\frac{\partial C_{g,m+1}}{\partial z}\right|_{z=0,t} \; (m = 1, 2, 4, 5, 6) \\
-\mathcal{D}_g \left.\frac{\partial C_{g,m}}{\partial z}\right|_{z=L_a,t} \pm V_m \, C_{g,m}\big|_{z=L_a,t} &= -2\mathcal{D}_g \left.\frac{\partial C_{g,m+1}}{\partial z}\right|_{z=0,t} \; (m = 3) \\
\left.\frac{\partial C_{g,7}}{\partial z}\right|_{z=L_a,t} &= 0
\end{aligned} \tag{3.2}$$

In these equations, z is the axial coordinate in an airway, oriented in the direction of the alveolar sacs, with z equal to zero at the proximal end of the airway and equal to L_a at its distal end. $C_{g,m}(z,t)$ is the local NO concentration in the gas, at time t and at position z in an airway in generation m of the acinus. $\mathcal{D}_g$ is the diffusion coefficient of NO in the lumen (its value for the diffusion of NO in air is given in Table 2). The convection-diffusion equation (Equation 3.1) is composed of three terms. The term on the left-hand side is the convection term. In this term, the $\pm$ sign accounts for the direction of the gas flow during inspiration $(+)$ or expiration $(-)$, and V_m is the average axial velocity of the gas in generation m of the acinus. For $m = 1, 2$ and 3, $V_m = Q_a(t)/2^{m-1}$, while V_m is set to zero in the last four generations, to mimic the fact that they inflate during inspiration or deflate during expiration. The right-hand side is composed of a diffusion term and a term involving the exchange flux density defined in Equation 13. The boundary conditions (Equations 3.2) describe the continuity of the NO concentration at the interface between two consecutive generations, the conservation of the NO flux at the interface between two consecutive generations and the absence of an axial diffusive flux at the distal end of the last generation in the acinus.

The system of equations composed of 3.1 and 3.2 can be solved analytically (quite easily with Wolfram Mathematica), in order to provide a relation (not explicitly given here due to its prohibitive size) between

the concentration gradient at the proximal end inlet of an acinus, $\partial C_{g,1}/\left.\partial z\right|_{z=0,t}$, the perfusion coefficient of the acinus γ (see Equation 13), and the NO concentration and gas flow rate at this proximal end:

$$\left.\frac{\partial C_{g,1}}{\partial z}\right|_{z=0,t} = f^{\pm}\left(\gamma, C_a(t), Q_a(t)\right) \tag{3.3}$$

The sign $\pm$ as a superscript accounts for the fact that two versions of the function f exist, whether inspiration or expiration is considered (+ or - sign on the left-hand side member of Equation 3.1). As mentioned in the core of the paper (see section 2.3.2), Equation 3.3 is a connecting link between the model of the NO transport in an acinus and the model of the NO transport in the tracheobronchial tree. Indeed, it is included in a boundary condition written at the end of each terminal airway. Note that, in the Wolfram Mathematica file, due to the complexity of the analytical expression for $f^{\pm}$, it is an approximate polynomial expression that is used in these boundary conditions. For each simulation, the constants in this polynomial approximation are identified in order to minimize the difference, in the least square sense, between the polynomial approximation and the analytical expression of $f^{\pm}$.

APPENDIX 4 - NUMERICAL SCHEME FOR THE UNSTEADY EQUATIONS

In this appendix, we propose a numerical scheme to solve the time-dependent system formed by Equation 15 and Equations 16a to 16c presented in the article. In the following, we write $C_{i,j}(t)$ the NO concentration at the center of the airway (i, j) at time t. The numerical scheme is presented here in the case of an expiration and for j being even, but it can be easily extended to any other situation. The numerical scheme is based on the finite volume method. A set of ordinary differential equations is written to express the time evolution of $C_{i,j}(t)$, $\forall(i, j) \in \mathcal{A}$. These equations are deduced from a mass balance on each of the airways in the tracheobronchial tree. These equations are different whether (i, j) refers to an intermediate ($(i, j) \in \mathcal{B}$) or to a terminal airway ($(i, j) \in \mathcal{T}$).

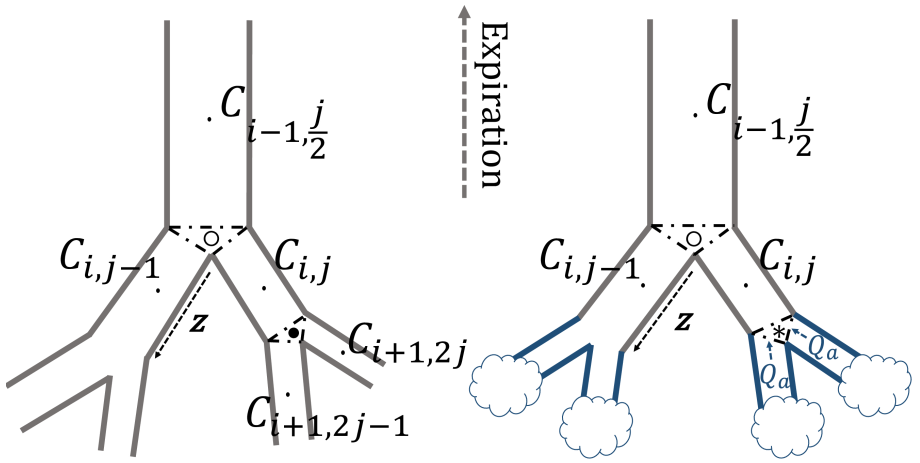

Figure 4.1. Schematic representation of two intermediate airways, (i, j) and $(i, j-1)$, located in a 3 generation structure of the tracheobronchial tree (left) and of two terminal airways, (i, j) and $(i, j-1)$, together with their two acini (represented in blue) (right).

For an intermediate airway, the following equation is proposed:

$$
\begin{aligned}
\frac{\pi D_{i,j}^3 \alpha_i}{4}\frac{dC_{i,j}}{dt} &= Q_{i+1,2j}C_{i+1,2j} + Q_{i+1,2j-1}C_{i+1,2j-1} - Q_{i,j}C_{i,j} \\
&+ \mathcal{D}_g \frac{\pi D_{i,j}^2}{4}\frac{C_\circ - C_{i,j}}{L_{i,j}/2} - \mathcal{D}_g \frac{\pi D_{i,j}^2}{4}\frac{C_{i,j} - C_\bullet}{L_{i,j}/2}
\end{aligned}
\tag{4.1}
$$

where $C_\bullet$ is the NO concentration at the division of the airway (i, j) into the airways $(i + 1, 2j)$ and $(i + 1, 2j - 1)$, and $C_\circ$ is the NO concentration at the division of the airway $(i - 1, j/2)$ into the airways (i, j) and $(i, j - 1)$ (see Figure 4.1).

The first three terms of the right-hand side of Equation 4.1 express the amounts of NO entering and leaving, per unit of time, the airway (i, j) by convection. The next two terms express the amounts of NO entering and leaving, per unit of time, the airway (i, j) through back-diffusion.

At any time, $C_\bullet$ and $C_\circ$ are evaluated with Equations 4.2 and 4.3, expressing the conservation of the diffusion fluxes at the bifurcations.

$$
\mathcal{D}_g \frac{\pi D_{i,j}^2}{4}\frac{C_{i,j} - C_\bullet}{L_{i,j}/2} = \mathcal{D}_g \frac{\pi D_{i+1,2j-1}^2}{4}\frac{C_\bullet - C_{i+1,2j-1}}{L_{i+1,2j-1}/2} + \mathcal{D}_g \frac{\pi D_{i+1,2j}^2}{4}\frac{C_\bullet - C_{i+1,2j}}{L_{i+1,2j}/2}
\tag{4.2}
$$

$$
\mathcal{D}_g \frac{\pi D_{i-1,j/2}^2}{4}\frac{C_{i-1,j/2} - C_\circ}{L_{i-1,j/2}/2} = \mathcal{D}_g \frac{\pi D_{i,j}^2}{4}\frac{C_\circ - C_{i,j}}{L_{i,j}/2} + \mathcal{D}_g \frac{\pi D_{i,j-1}^2}{4}\frac{C_\circ - C_{i,j-1}}{L_{i,j-1}/2}
\tag{4.3}
$$

Equation 4.3 cannot be used for the trachea (airway $(1, 1)$). For this airway, the diffusion flux at the proximal end of the airway (i.e. at the top of the trachea) is set to zero: $C_\circ = C_{1,1}$.

For a terminal airway, the following equation is proposed:

$$
\frac{\pi D_{i,j}^3 \alpha_i}{4}\frac{dC_{i,j}}{dt} = Q_{i,j}(C_* - C_{i,j}) + \mathcal{D}_g \frac{\pi D_{i,j}^2}{4}\frac{C_\circ - C_{i,j}}{L_{i,j}/2} - \mathcal{D}_g \frac{\pi D_{i,j}^2}{4}\frac{C_{i,j} - C_*}{L_{i,j}/2}
\tag{4.4}
$$

where C_* is the NO concentration at the location of the division of the airway (i, j) into its two "daughter" acini, and $C_\circ$ is the NO concentration at the location of the division of the airway $(i - 1, j/2)$ into the airways (i, j) and $(i, j - 1)$ (see Figure 4.1). $C_\circ$ is evaluated using Equation 4.3 and C_* is evaluated, at any time, using Equation 4.5, expressing the conservation of the diffusion flux at the bifurcation between airway (i, j) and the two subsequent acini (using the function f^- defined in Equation 3.3).

$$
\mathcal{D}_g \frac{\pi D_{i,j}^2}{4}\frac{C_{i,j} - C_*}{L_{i,j}/2} = 2\mathcal{D}_g \frac{\pi D_a^2}{4} f^-(C_*, \frac{Q_{i,j}}{2})
\tag{4.5}
$$

To assess the quality of this numerical scheme, it is used to fully simulate a F_ENO test and the results at the end of the slow expiration at 50 ml/s (during 10 s) are compared to the results obtained with the analytical resolution of the steady equations (described in Section 2.3 of the article). Conditions where $\phi_{i,j} = 0.1$ for 50% of the terminal airways, starting from the ones closest to the trachea, are used and the comparison is

shown in Figure 4.2. We observe a good agreement between the results of the two methods. Additionally, we have observed that the computational time for the resolution based on the numerical scheme is much shorter than the computational time required for the analytical steady resolution.

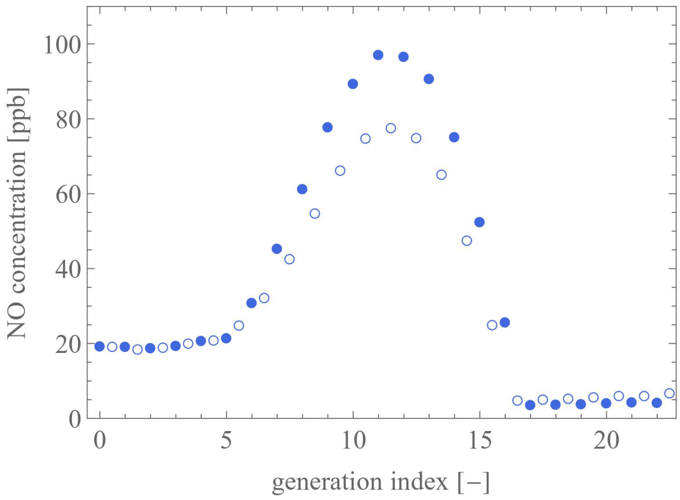

Figure 4.2. Average NO concentration profile at the end of a 10 s expiration at a flow rate of 50 ml/s, where $\phi_{i,j}$ is set to 0.1 for 50% of the terminal airways (the ones closest to the trachea). The filled blue circles show the NO concentration profile obtained using the analytical resolution whereas the empty blue circles show the NO concentration profile obtained with the time-dependent numerical scheme.

REFERENCES

[1]C. Karamaoun, A. Van Muylem, and B. Haut. Modeling of the nitric oxide transport in the human lungs. *Frontiers in Physiology*, 7:255, 2016.

[2]M. Paiva. Computation of the boundary conditions for diffusion in the human lung. *Computers and Biomedical Research*, 5:585–595, 1972.

[3]M. Paiva. Gas transport in the human lung. *Journal of Applied Physiology*, 35(3):401–410, 1973.

[4]T. J. Pedley, R. C. Schroter, and M. F. Sudlow. Energy losses and pressure drop in models of human airways. *Respiration Physiology*, 9(3):371–386, 1970.

[5]T. J. Pedley, R. C. Schroter, and M. F. Sudlow. The prediction of pressure drop and variation of resistance within the human bronchial airways. *Respiration Physiology*, 9(3):387–405, 1970.
